# Molecular dissection of maize seedling salt tolerance using a genome‐wide association analysis method

**DOI:** 10.1111/pbi.13607

**Published:** 2021-05-02

**Authors:** Meijie Luo, Yunxia Zhang, Jingna Li, Panpan Zhang, Kuan Chen, Wei Song, Xiaqing Wang, Jinxiao Yang, Xiaoduo Lu, Baishan Lu, Yanxin Zhao, Jiuran Zhao

**Affiliations:** ^1^ Beijing Key Laboratory of Maize DNA Fingerprinting and Molecular Breeding Maize Research Center Beijing Academy of Agriculture and Forestry Sciences (BAAFS) Beijing China; ^2^ Institute of Molecular Breeding for Maize Qilu Normal University Jinan China

**Keywords:** salt tolerance, genetic loci, association mapping, maize

## Abstract

Salt stress is a major devastating abiotic factor that affects the yield and quality of maize. However, knowledge of the molecular mechanisms of the responses to salt stress in maize is limited. To elucidate the genetic basis of salt tolerance traits, a genome‐wide association study was performed on 348 maize inbred lines under normal and salt stress conditions using 557 894 single nucleotide polymorphisms (SNPs). The phenotypic data for 27 traits revealed coefficients of variation of >25%. In total, 149 significant SNPs explaining 6.6%–11.2% of the phenotypic variation for each SNP were identified. Of the 104 identified quantitative trait loci (QTLs), 83 were related to salt tolerance and 21 to normal traits. Additionally, 13 QTLs were associated with two to five traits. Eleven and six QTLs controlling salt tolerance traits and normal root growth, respectively, co‐localized with QTL intervals reported previously. Based on functional annotations, 13 candidate genes were predicted. Expression levels analysis of 12 candidate genes revealed that they were all responsive to salt stress. The CRISPR/Cas9 technology targeting three sites was applied in maize, and its editing efficiency reached 70%. By comparing the biomass of three CRISPR/Cas9 mutants of *ZmCLCg* and one *zmpmp3* EMS mutant with their wild‐type plants under salt stress, the salt tolerance function of candidate genes *ZmCLCg* and *ZmPMP3* were confirmed. Chloride content analysis revealed that *ZmCLCg* regulated chloride transport under sodium chloride stress. These results help to explain genetic variations in salt tolerance and provide novel loci for generating salt‐tolerant maize lines.

## Introduction

Soil salinization is a globally devastating environmental problem resulting from both natural and artificial processes, such as mineral weathering and irrigation. Salinity affects more than 800 million hectares of land, accounting for more than 6% of the world’s land area (Munns and Tester, 2008). Various types of ions are responsible for salination, including sodium, potassium, calcium, magnesium and chloride (Zhu, 2016). Sodium chloride (NaCl) is the most abundant salt in soil because of its high solubility and ubiquitous distribution (Munns and Tester, 2008). Soil salinity has many negative effects on plant growth and development, including inhibition of seed germination, reduced root growth, plant height and fruiting levels, which ultimately decreases crop yield and quality (Sandhu *et al*., 2020). More seriously, sensitive crops can be killed by even slight salinity in soils (Luo *et al*., 2019b). The bases of these phenomena have been dissected using molecular genetics analyses. Such analyses have shown that various physiological and metabolic functions in plants are impaired by osmotic, ionic and oxidative stresses under saline conditions (Muchate *et al*., 2016).

Because of the harmful effects of salinity stress on plant growth and crop yields, it is necessary to exploit the genetic basis of variability in salt tolerance to improve plants’ resistance to salt toxicity. Quantitative trait locus (QTL) mapping has revealed many genomic regions that affect important traits. In maize, QTL analyses of the germination rate, salt tolerance ranking, shoot fresh and dry weights, shoot K^+^/Na^+^ ratio and Na^+^ and K^+^ concentrations in shoots were conducted on seedlings of 161 F_2:5_ lines under hydroponic culture and saline field conditions. In total, 38 salt tolerance‐related QTLs were detected on chromosomes 1, 3 and 5, with eight being major QTLs that individually explained more than 20% of the phenotypic variation (Cui *et al*., 2015). Using plant height in a saline field and a plant height‐based salt tolerance index (plant height in a saline field/plant height in a normal field) as salt tolerance indicators, QTLs were detected in mature field‐grown maize plants of 240 double‐haploid lines. A major QTL, *qSPH1*, which was responsible for the two salt tolerance‐related traits, was identified on chromosome 1 and explained 25.9%–31.2% of the phenotypic variation (Luo *et al*., 2017b). Some QTLs associated with salt tolerance have also been identified in other crops, such as rice (Zeng *et al*., 2021), wheat (Luo *et al*., 2021), soybean (Guan *et al*., 2014) and tomato (Frary *et al*., 2010). The results of those QTL mapping studies have provided insights into the genetic mechanisms of plant salt tolerance, which may be useful for breeding salt‐tolerant crops.

A number of functional genes and transcriptional factors related to salt tolerance have been reported. Many of them are involved in ion homeostasis maintenance, osmotic protection, antioxidant regulation, hormonal regulation and Ca^2+^ signalling pathways (Muchate *et al*., 2016). For example, in the model plant Arabidopsis, the salt overly sensitive (SOS) signalling pathway (comprising *SOS1*, *SOS2* and *SOS3*) involved in salt tolerance has been well characterized (Zhu, 2002; Zhu, 2016). In this pathway, SOS3 senses the Ca^2+^ signal elicited by ion stress, and then interacts with SOS2. The activated SOS2 phosphorylates the plasma membrane Na^+^/H^+^ antiporter SOS1, and the activated SOS1 extrudes Na^+^ from the cytosol (Zhu, 2016). In rice, the *SKC1* gene, which encodes a high affinity K^+^ transporter (HKT), has been successfully isolated through map‐based cloning. Overexpression of *SKC1* was shown to reduce the Na^+^ content and increase the K^+^ content in the shoots of salt‐stressed rice (Ren *et al*., 2005). In maize, *ZmHKT1*, which also encodes an HKT‐type transporter, has been isolated from the major maize salt tolerance QTL, *ZmNC1* (logarithm of odds, LOD = 12.51). A gene knock‐out analysis verified that *ZmHKT1* is important for Na^+^ homeostasis and salt tolerance in maize (Zhang *et al*., 2018). However, despite the dissection of numerous genetic loci, the molecular basis of salt tolerance in plants is still far from being completely understood.

A genome‐wide association study (GWAS) is a powerful method to study genetic variations associated with complex traits at the genome‐wide level (Huang *et al*., 2010; Luo *et al*., 2019b; Xie *et al*., 2019). This method takes into account the historical recombination found in broad panels of diverse germplasm and population‐wide linkage disequilibrium (LD) among single nucleotide polymorphisms (SNPs) and QTLs. Thus, a GWAS can circumvent the limiting low recombination rate of bi‐parental populations to identify a wider range of genetic variations and provide complementary information (Lu *et al*., 2018). Recently, GWAS technology has been used to dissect multiple complex trait‐related mechanisms in various plant species. In rice, a metabolic GWAS identified 36 candidate genes that modulate the levels of physiologically and nutritionally important metabolites (Chen *et al*., 2014). In soybean, a GWAS led to the identification of 11 candidate genes related to days to flowering, maturity and plant height (Zhang *et al*., 2015). Additionally, using a GWAS, the natural variation of *ZmVPP1* encoding one vacuolar‐type H^+^ pyrophosphatase was identified, which contributed significantly to drought tolerance of maize (Wang *et al*., 2016). Four stable QTLs were identified in maize, which played critical roles in controlling arsenic accumulation (Zhao *et al*., 2018).

Maize is an important cereal crop worldwide and is also moderately salt sensitive. In general, the germination and seedling phases of maize are more sensitive than other phases to salt stress (Luo *et al*., 2019a; Luo *et al*., 2017a). In previous studies, researchers have identified salt stress‐related QTLs such as *qRLS1* (Luo *et al*., 2019a) and *QFgr1* (Cui *et al*., 2015), and a few genes regulating ion transport and gene transcription, such as *ZmHAK4* (Zhang *et al*., 2019), *ZmHKT1* (Zhang *et al*., 2018), *ZmPMP3* (Fu *et al*., 2012), *ZmbZIP72* (Ying *et al*., 2012), *ZmMPK5* (Zhang *et al*., 2014), *Zmhdz10* (Zhao *et al*., 2014), *ZmSIMK1* (Gu *et al*., 2010), *SAG4* (Luo *et al*., 2019b) and *SAG6* (Luo *et al*., 2019b). However, the molecular mechanisms of salt tolerance in maize are poorly understood.

In the present study, a high‐density SNP‐based GWAS analysis was performed under normal and salt stress conditions to detect natural variations in alleles related to the salt stress response during the maize germination stage. The main purpose of this study was to investigate significant alleles and potential candidate genes associated with shoot and root traits related to salt tolerance. The findings of this study shed light on the molecular mechanisms associated with variations in salt tolerance among maize inbred lines and may assist in the development of molecular markers for the improvement of salt tolerance in maize.

## Results

### Phenotypic variation analysis

The maize diversity panel consisting of 348 accessions collected from the USA, China, and CIMMYT has been used for several GWAS analyses (Li *et al*., 2013; Liu *et al*., 2017; Yang *et al*., 2011b) (Table S1). In this study, phenotypic data for nine important growth‐related traits [shoot length (SL), root length (RL), full length of seedling (FL), shoot fresh weight (SF), root fresh weight (RF), full fresh weight of seedling (FF), shoot dry weight (SD), root dry weight (RD) and full dry weight of seedling (FD)], were collected from the association population under normal and salt‐stressed conditions. The salt tolerance indexes of these nine traits were calculated by dividing the values measured under salt stress conditions by the values measured under control conditions. The phenotypic frequencies of all 27 traits (nine traits under normal conditions and salt‐stressed conditions, and the salt tolerance indexes of these traits) exhibited normal or near‐normal distributions (Figure S1). The average values of SL, RL, FL, SF, RF, FF, SD, RD and FD were 4.03 cm, 5.83 cm, 9.87 cm, 0.12 g, 0.18 g, 0.30 g, 0.018 g, 0.02 g and 0.04 g, respectively, under salt stress conditions, compared with 11.10 cm, 11.27 cm, 22.37 cm, 0.35 g, 0.33 g, 0.67 g, 0.04 g, 0.03 g and 0.07 g, respectively, under control conditions. The average values of the salt tolerance indexes (R) of the nine traits were as follows: SL (SLR = 0.38), RL (RLR = 0.57), FL (FLR = 0.47), SF (SFR = 0.36), RF (RFR = 0.56), FF (FFR = 0.46), SD (SDR = 0.46), RD (RDR = 0.64) and FD (FDR = 0.54). All 27 traits showed a wide range of phenotypic variation, and their coefficients of variation were all greater than 25%. The repeatability for all traits was high (74.31%–97.26%) (Table 1).

**Table 1:** Phenotypic variations of traits in the maize association population

Trait	Range	Mean ± SD	CV (%)	Skewness	Kurtosis	Repeatability (%)
SL	2.782–20.890 cm	11.10 ± 3.63	32.73	0.38	−0.44	94.91
RL	3.625–34.360 cm	11.27 ± 5.72	50.72	1.19	0.67	97.26
FL	8.080–53.260 cm	22.37 ± 8.68	38.79	0.91	0.11	97.05
SF	0.092–0.803 g	0.35 ± 0.13	37.29	0.63	0.22	94.78
RF	0.076–0.832 g	0.33 ± 0.13	38.91	0.76	0.68	95.60
FF	0.168–1.635 g	0.67 ± 0.24	35.90	0.67	0.41	95.42
SD	0.009–0.083 g	0.04 ± 0.01	27.49	0.35	0.21	92.06
RD	0.006–0.069 g	0.03 ± 0.01	28.71	0.24	0.42	92.92
FD	0.016–0.152 g	0.07 ± 0.02	25.48	0.25	0.53	92.99
SLS	1.150–9.460 cm	4.03 ± 1.45	36.03	0.78	0.72	92.04
RLS	2.130–12.930 cm	5.83 ± 2.01	34.42	0.88	0.64	94.77
FLS	4.040–22.390 cm	9.87 ± 3.15	31.96	0.73	0.43	95.00
SFS	0.034–0.298 g	0.12 ± 0.05	40.41	0.70	0.51	90.86
RFS	0.026–0.469 g	0.18 ± 0.08	46.42	0.85	0.66	92.80
FFS	0.075–0.699 g	0.30 ± 0.12	41.09	0.75	0.34	92.97
SDS	0.005–0.036 g	0.018 ± 0.01	34.92	0.30	−0.38	90.71
RDS	0.001–0.047 g	0.02 ± 0.01	36.06	0.11	0.33	90.72
FDS	0.006–0.082 g	0.04 ± 0.01	31.65	0.18	0.21	91.20
SLR	0.134–1.114	0.38 ± 0.13	34.21	1.35	4.63	84.45
RLR	0.166–1.594	0.57 ± 0.18	31.40	1.07	3.66	81.35
FLR	0.182–1.061	0.47 ± 0.13	27.40	1.00	2.25	82.96
SFR	0.127–0.912	0.36 ± 0.12	33.52	0.86	1.54	77.04
RFR	0.097–1.611	0.56 ± 0.18	32.76	0.88	3.18	74.66
FFR	0.153–1.038	0.46 ± 0.13	28.72	0.74	1.47	74.31
SDR	0.176–1.094	0.46 ± 0.14	29.40	0.74	1.80	76.80
RDR	0.048–1.892	0.64 ± 0.20	31.14	0.61	4.31	77.53
FDR	0.145–1.259	0.54 ± 0.14	25.22	0.54	2.91	74.88

**Figure 1:**
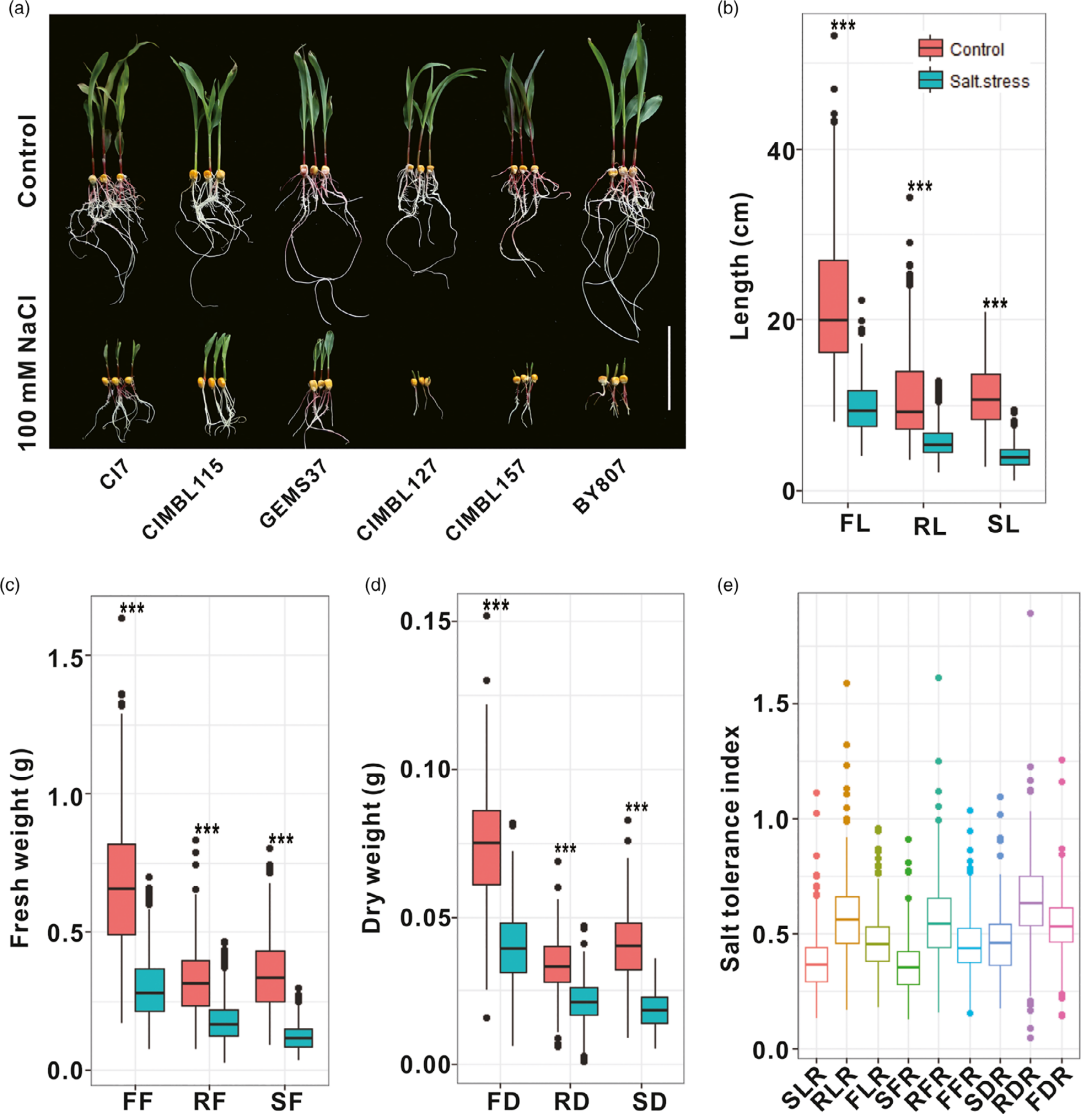
Distribution of measured traits in maize under control and salt stress conditions. (a) Growth status of three significantly salt tolerant (CI7, CIMBL115 and GEMS37) and three significantly salt sensitive (CIMBL127, CIMBL157 and BY807) maize inbred lines. Bar = 10 cm; (b) Seedling length; (c) Seedling fresh weight; (d) Seedling dry weight; (e) Salt tolerance index. Data for each trait are mean values of three biological replicates of maize association population. ***Significant at *P* < 0.001. SL: shoot length, RL: root length, FL: full length of seedling, SF: shoot fresh weight, RF: root fresh weight, FF: full fresh weight of seedling, SD: shoot dry weight, RD: root dry weight, FD: full dry weight of seedling. SL, RL, FL, SF, RF, FF, SD, RD and FD represent traits under normal conditions; SLS, RLS, FLS, SFS, RFS, FFS, SDS, RDS and FDS represent traits under salt stress conditions; SLR, RLR, FLR, SFR, RFR, FFR, SDR, RDR and FDR represent salt tolerance indexes of traits.

The phenotypes of three significantly salt tolerant (CI7, CIMBL115 and GEMS37) and three significantly salt sensitive (CIMBL127, CIMBL157 and BY807) maize inbred lines were shown in Figure 1a. In general, the nine measured traits (SL, RL, FL, SF, RF, FF, SD, RD and FD) were significantly lower (35.6%–63.7% decrease, *P* < 0.001) under salt treatment conditions than under control conditions. The SL, SF and SD showed greater decreases than RL, RF and RD in response to salt stress, consistent with previous reports (Luo *et al*., 2017a; Luo *et al*., 2018) (Figure 1b–e). The nine measured traits of seedlings were significantly and positively correlated with each other (*P* < 0.001) under salt stress and control conditions. As expected, the salt tolerance indexes of the nine traits were negatively correlated with the nine traits under control conditions. The correlation coefficients of the traits under the same conditions were relatively higher than the correlation coefficients of the traits between different conditions. Furthermore, the correlation coefficients between traits under salt and control conditions were the greatest in all correlation coefficients among traits under salt treatment, under control treatment and their salt tolerance indexes (Figure S2).

### GWAS mapping

In total, more than 1.06 million high‐quality SNPs were obtained from an RNA‐seq project and the MaizeSNP50 BeadChip (Fu *et al*., 2013; Ganal *et al*., 2011). The SNPs were filtered with a minor allele frequency <0.05, and the remaining 557 894 SNPs were used for the association analysis. To determine the optimal model for the association analysis, three models (K, Q, Q + K) were compared using quantile–quantile plots (Figure S3). The Q and Q + K models resulted in a greater control of false‐negative errors, and the Q + K model was more reliable than the Q model. Moreover, by combining the two covariates of population structure (Q) and K, the Q + K method can effectively control type I errors (false positives) (Lu *et al*., 2018; Zhang *et al*., 2016). Therefore, all subsequent GWAS analyses were performed using the Q + K model. Manhattan plots for all traits are shown in Figure S4. The quantile–quantile (QQ) plots for all traits were shown in Figure S5.

**Figure 2:**
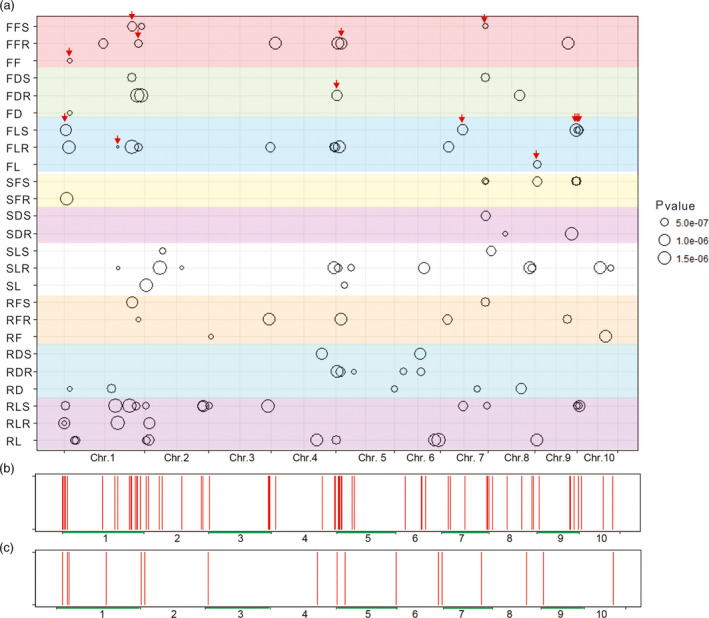
Chromosomal distribution of quantitative trait loci (QTL) for maize traits under control and salt stress conditions. (a) Chromosomal distribution of salt tolerance‐associated and growth‐related QTLs in maize. QTLs are represented by black circles and circle size indicates significance of association. Red arrows show QTLs associated with multiple traits. (b) Chromosomal distribution of salt tolerance‐associated QTLs in maize. QTLs are represented by red vertical lines. (c) Chromosomal distribution of normal growth‐related QTLs in maize. QTLs are represented by red vertical lines.

A total of 183 SNP‐trait associations with *P* < 1.79 × 10^−6^ were identified (Table S2), and they involved 149 unique SNPs. According to the LD decay distance of this maize population, a 200‐kb region (±100 kb) around each significant SNP was defined as a QTL (Deng *et al*., 2017; Wang *et al*., 2019). The QTLs with overlapping intervals for the same traits were merged. In this way, we identified 83 QTLs related to salt stress and 21 QTLs in the control, with an average of 4.6 and 3.0 loci for each trait, respectively (Table S3; Figure 2a‐c). Briefly, 36 QTLs (42 significant SNPs) were identified for the nine traits under salt treatment conditions, and the proportion of phenotypic variation (*R* ^2^) explained by each locus ranged from 6.9% to 11.2%, with a mean of 7.6%. In the control, 21 QTLs (29 significant SNPs) were identified for seven traits, and the proportion of phenotypic variation explained by each locus ranged from 6.7%–9.7%, with an average of 7.6%. Based on the salt tolerance indexes of the nine traits, 47 QTLs (78 significant SNPs) were identified, with *R* ^2^ values ranging from 6.6%–10.5% (average, 7.2%).

The co‐localization of QTLs identified from multiple traits was summarized (Figure 2; Table S4). Overall, 11 QTL intervals were simultaneously detected by multiple salt‐related traits (Figure 2a,b), while two QTL intervals were simultaneously detected by multiple traits in the control (Figure 2a,c). The chromosomal distribution of salt‐associated QTLs revealed a hot spot on chromosome 5 (4.56–19.35 Mb) (Figure 2b). These QTLs and the hot spot might play an important role in regulating salt tolerance in maize.

### Candidate gene prediction and expression profiling

The physical positions of the 83 salt‐related QTLs from the maizeGDB database (www.maizegdb.org, B73 RefGen_v2) were searched to identify the genes present within these QTL regions. A total of 420 genes were obtained. Based on their annotations in the maizeGDB, Gramene and TAIR databases, 16 genes were found to be involved in tolerance to salinity or water deficiency. By analysing the LD of significant SNPs associated with candidate genes, we found that three genes were outside the LD regions of their significant SNPs (*r^2^* < 0.1). Therefore, they were excluded from the candidate genes. The remaining 13 genes were considered as candidate genes (Table 2).

**Table 2:** Candidate maize salt tolerance‐related genes revealed by functional annotations

Traits	Peak SNP	Allele	Chr.	QTL interval (bp)	*P* value	*R*^2^ (%)	Candidate gene	Position (bp)	Annotation	Ortholog	Ortholog annotation
RFS, FDS, FFS	PZE‐101201482	A/G	1	252 259 061–252 459 061	5.65E‐07	11.15	GRMZM2G121570	252 320 123–252 322 173	myb73, MYB transcription factor 73, regulation of transcription		
RL, SLR, FLR	chr1.S_201957459	C/T	1	201 857 459–202 057 459	2.06E‐09	10.51	GRMZM2G028386	201 957 089–201 958 672	ereb137, AP2‐EREBP transcription factor 137	AT2G40340	DREB2C, encodes a member of the DREB subfamily A‐2 of ERF/AP2 transcription factor family, response to drought
RLS	chr2.S_5846479	A/G	2	5 739 326–5 947 477	3.45E‐07	7.86	GRMZM2G071119	5 897 689–5 904 448	‐‐‐	AT5G33280	ATCLCG, voltage‐gated chloride channel family protein, chloride transmembrane transport
FLR	chr4.S_235409430	C/G	4	235 299 568–235509 431	6.17E‐07	7.16	GRMZM2G421857	235 439 768–235 449 150	vpp3, vacuolar proton pump3	AT1G78900	Encodes catalytic subunit A of the vacuolar ATP synthase, response to salt stress
FLR	chr5.S_12267893	C/T	5	12 167 893–12 367 893	1.294E‐06	6.73	GRMZM2G102754	12 267 646–12 273 705	ufg2, ethylene‐insensitive protein 2	AT5G03280	ATEIN2, involved in ethylene signal transduction, response to salt stress
SLR	chr5.S_6438730	G/C	5	6 338 730–6 538 731	4.92E‐07	7.40	GRMZM2G176085	6 357 462–6 359 213	‐‐‐	AT2G18980	Peroxidase superfamily protein, response to oxidative stress
RDR, FDR	chr5.S_4665179	T/G	5	4 565 179–4 765 179	9.07E‐07	7.09	GRMZM2G027351	4 651 173–4 655 150	cdpk18, calcium‐dependent protein kinase18		
RDR, FDR	chr5.S_4665179	T/G	5	4 565 179–4 765 179	9.07E‐07	7.09	GRMZM2G166049	4 570 654–4 574 357	mab17, math‐btb17, btb/poz domain protein	AT3G03740	ATBPM4, encodes a member of the MATH‐BTB domain proteins (BPMs) that directly interact with and target for proteasomal degradation the class I homeobox‐leucine zipper (HD‐ZIP) transcription factor ATHB6, cellular response to salt stress
RLS	chr7.S_174340400	G/T	7	174 240 400–174 440 400	3.25E‐07	7.89	GRMZM2G041636	174 327 890–174 328 868	‐‐‐	AT3G06590	Encodes RITF1, a bHLH transcription factor that regulates the transcription of several genes involved in the detoxification of reactive oxygen species generated by salt stress
SDS, SFS, FDS, RFS, FFS	chr7.S_168405013	A/G	7	168 304 086–168 505 013	9.99E‐08	8.58	GRMZM2G477325	168 401 590–168 402 719	pmpm5, proteolipid membrane potential regulator5. Plant Wide Gene Name: PMP3, encodes plasma membrane proteolipid involved in ion homeostasis and response to salinity		
SLR	chr8.S_163285326	G/T	8	163 185 326–163 385 326	4.59E‐07	7.44	GRMZM2G066024	163 307 256–163 309 969	ald2, aldolase	AT2G36460	Aldolase superfamily protein, response to salt stress
FDR	chr8.S_120609841	T/C	8	120 509 841–120 709 841	9.81E‐07	7.04	GRMZM2G033230	120 608 901–120 610 664	bzip108, bZIP transcription factor 108	AT3G51960	ATBZIP24, bZIP transcription factor, induced by salt stress and promoted salt tolerance
RLS	chr10.S_8805555	G/A	10	8 705 555–8 948 948	3.57E‐07	7.83	GRMZM2G136910	8 848 146–8 849 526	aasr1, abscisic acid stress ripening1, protects kernel yield under water deficit		

*GRMZM2G477325*, which encodes a plasma membrane protein 3 (PMP3) on chromosome 7, was within the QTL simultaneously detected by five salt‐associated traits (Table S4). This gene is known to be involved in ion homeostasis in yeast mutant under salt stress (Fu *et al*., 2012). Another gene, *GRMZM2G071119* (*ZmCLCg*), located on chromosome 2, encodes an unknown protein. However, its Arabidopsis ortholog is a voltage‐gated chloride channel that participates in chloride transmembrane transport (Nguyen *et al*., 2016), indicating that it is a likely candidate gene for chloride transport in maize.

Four candidate genes (*GRMZM2G102754*, *GRMZM2G176085*, *GRMZM2G027351* and *GRMZM2G166049*) located within the hot spot on chromosome 5 (4.56–19.35 Mb) (Figure 2b). The Arabidopsis orthologs of *GRMZM2G102754* and *GRMZM2G176085* play roles in ethylene signal transduction (Zhang *et al*., 2017) and oxidative stress responses (Valério *et al*., 2004) under salt stress, respectively. *GRMZM2G027351* and *GRMZM2G166049* encode a calcium‐dependent protein kinase 18 (cdpk18) (Mittal *et al*., 2017) and a btb/poz domain protein (mab17, math‐btb17) (Juranić *et al*., 2012), respectively. The Arabidopsis ortholog of *GRMZM2G166049* is involved in homeobox‐leucine zipper transcription factor degradation (Lechner *et al*., 2011).

Four other candidate genes (*GRMZM2G033230*, *GRMZM2G041636*, *GRMZM2G121570* and *GRMZM2G028386*) encode transcription factors and might be associated with salt tolerance through transcriptional regulation. The *GRMZM2G033230* encodes a bZIP transcription factor 108 (bZIP108) (Yilmaz *et al*., 2009); the Arabidopsis ortholog of *GRMZM2G041636* encodes a bHLH transcription factor (Guan *et al*., 2013); *GRMZM2G121570* encodes MYB transcription factor 73 (myb73) (Xiao *et al*., 2017) and *GRMZM2G028386* encodes AP2‐EREBP transcription factor 137 (ereb137) (Yilmaz *et al*., 2009).

In addition, *GRMZM2G136910* on chromosome 10 encodes an abscisic acid stress ripening 1 protein (aasr1), which was shown to improve maize kernel yield by regulating metabolic processes under water‐limited conditions (Virlouvet *et al*., 2011). Another gene, *GRMZM2G066024* on chromosome 8, encodes an aldolase (ald2) (Marocco *et al*., 2005), and its Arabidopsis ortholog responds to salt stress (Lu *et al*., 2012). *GRMZM2G421857* on chromosome 4 encodes vacuolar proton pump 3 (vpp3) (Viereck *et al*., 1996).

**Figure 3:**
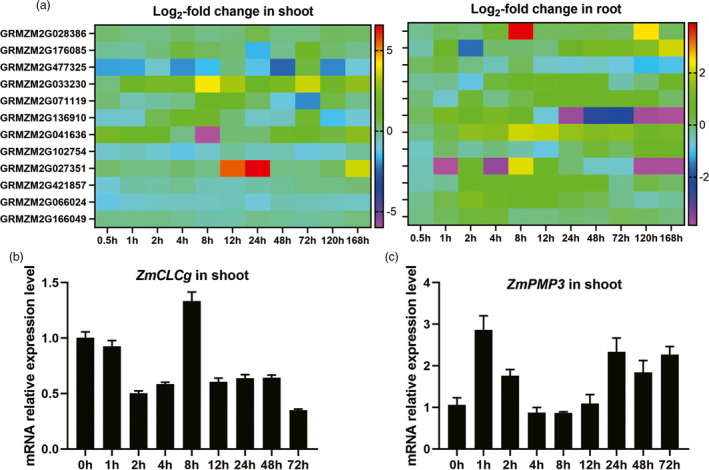
Transcript levels of candidate genes in shoots and roots of maize seedlings at different time points. (a) Fold changes in candidate genes’ transcript levels in shoots and roots of Jing724 seedlings at different time points compared with 0 h during salt treatment. Data represent means of fold changes from three biological replicates. (b and c) Transcript levels of *ZmCLCg* (b) and *ZmPMP3* (c) in shoots of B73 seedlings at different time points. Data were shown as the mean ± SE of three independent experiments.

The transcription levels of 12 candidate genes in root and shoot of maize inbred line Jing 724 at 0, 0.5, 1, 2, 4, 8, 12, 24, 48, 72, 120 and 168 h after salt treatment were obtained by RNA‐seq (among the 13 candidate genes, *GRMZM2G121570* was not detected in RNA‐seq). The expression of all 12 genes fluctuated from 0.5–168 h after salt treatment (Figure 3a). In shoot, except *GRMZM2G102754* and *GRMZM2G066024*, the expression of all genes was up‐regulated at certain time points. *GRMZM2G033230*, which encodes a bZIP transcription factor (Yilmaz *et al*., 2009), showed high transcript levels at all time points under salt treatment. In roots, the expression levels of all genes were up‐regulated at multiple time points (Figure 3a). These results indicated that all 12 candidate genes were responsive to salt stress.

The expression levels of *ZmCLCg* (*GRMZM2G071119*) and *ZmPMP3* (*GRMZM2G477325*) in shoot of B73 under salt stress were analysed. Results showed that *ZmCLCg* and *ZmPMP3* were responsive to salt stress in B73. In shoot, the expression of *ZmCLCg* was up‐regulated at 8 h (Figure 3b) while *ZmPMP3* was up‐regulated at 1 h post salt treatment (Figure 3c).

## Functional verification of candidate genes *ZmCLCg* and *ZmPMP3*

After culturing in control or saline water for 10 days, growth parameters of B104, B73, the significantly salt‐tolerant maize inbred line GEMS37, and the significantly salt sensitive maize inbred line CIMBL157 were compared (Figure S6a–e). The RL, RF, SL and SF of GEMS37, B104 and B73 were significantly higher than those of CIMBL157. The salt tolerance indexes of RL, RF, SL and SF in GEMS37 were 59.8%, 41.8%, 41.6% and 49.3%, respectively. In B104, they were 60.9%, 57.7%, 48.1% and 46.3%, respectively. In B73, they were 53%, 49.0%, 43.9% and 35.6%, respectively. In CIMBL157, they were 32.3%, 23.6%, 10.0% and 11.4%, respectively. The growth parameters under salt stress and the salt tolerance indexes of B104 and B73 were close to that of salt tolerant maize inbred line GEMS37 (Figure S6a–e). These results suggested that both B104 and B73 were salt tolerant maize inbred lines.

**Figure 4:**
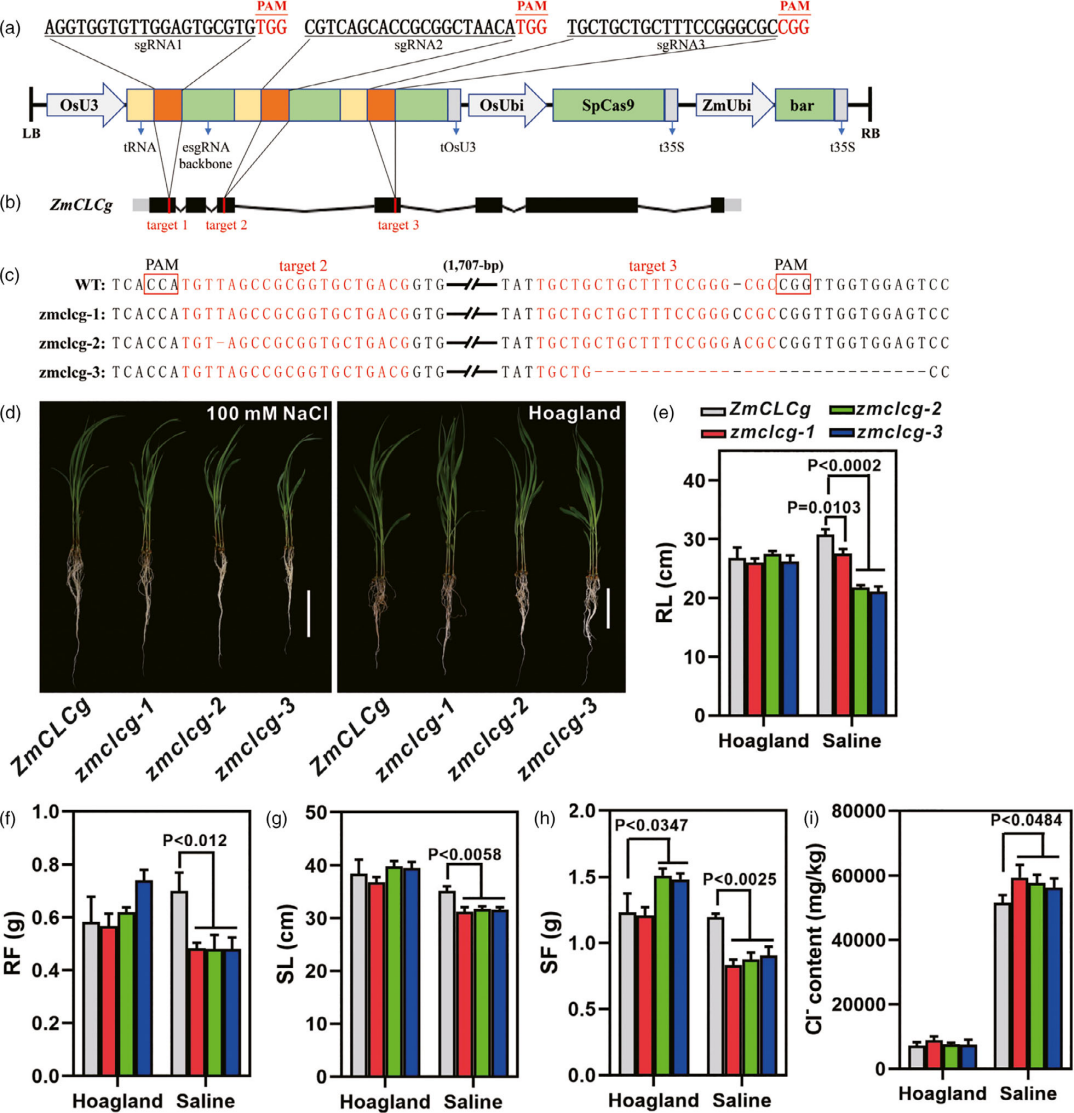
Growth parameters and chloride content of *ZmCLCg* CRISPR/Cas9 knock‐out mutants and their wild type (B104) under control and saline conditions. (a) Schematic illustration of the SpCas9 construct and three target sites (brown boxes). (b) Gene structure of *ZmCLCg* and the three target sites. (c) Sequences of target 2 and target 3 of *ZmCLCg* in wild type and three knock‐out mutants. (d) Growth morphologies of the wild type B104 and three *ZmCLCg* knock‐out maize lines after a 7‐day exposure to saline and control treatment. Bar = 10 cm. (e–h) Root length (RL) (e), root fresh weight (RF) (f), shoot length (SL) (g) and shoot fresh weight (SF) (h) of the wild type and three knock‐out mutants under control and saline conditions. (i) Chloride content in shoot. Data are shown as the mean ± SD of three independent experiments. The *P* values were calculated by a two‐tailed Student’s *t* test.

To validate the function of candidate gene *ZmCLCg* in salt tolerance, the maize inbred line B104 with *ZmCLCg* gene knock out was generated by CRISPR/Cas9 technology. The CRISPR/Cas9 vector used for *ZmCLCg* editing contains a synthetic polycistronic gene with three tandem tRNA‐gRNA structures, which has the advantage of producing multiple mature gRNAs through endogenous tRNA‐processing system (Xie *et al*., 2015) (Figure 4a). Three 20‐bp sequences in the first, third and fourth exons of *ZmCLCg* were chosen as Cas9‐gRNA cleavage sites (Figure 4a,b). PCR and sequencing analysis identified one mutant plant in the third exon (Mutation efficiency = 10%) and 7 mutant plants in the fourth exon (Mutation efficiency = 70%) among 10 independent T0 transgenic lines. These mutants were self‐pollinated to obtain homozygous mutant maize plants. Three *zmclcg* knock‐out mutants and the wild type were used to investigate their biomass parameters under salt stress. The z*mclcg‐1* has single base insertion mutation at the target site of exon 4, the *zmclcg‐2* has single base deletion at exon 3 and single base insertion at exon 4, and the *zmclcg‐3* has 28‐bp deletion mutation at the target site of exon 4 (Figure 4c). These mutations will lead to frame shift mutations in *ZmCLCg* gene. All three *zmclcg* mutants showed a greater reduction in root length, root fresh weight, shoot length and shoot fresh weight compared with that of the wild type under 100 mm NaCl treatment (Figure 4d–h). These results suggested that *ZmCLCg* conferred salt tolerance in maize. Under 100 mm NaCl treatment, the chloride content in shoots of three *zmclcg* mutants was significantly higher than that of wild type (Figure 4i), indicating that the salt tolerance function of ZmCLCg was associated with chloride transport.

**Figure 5:**
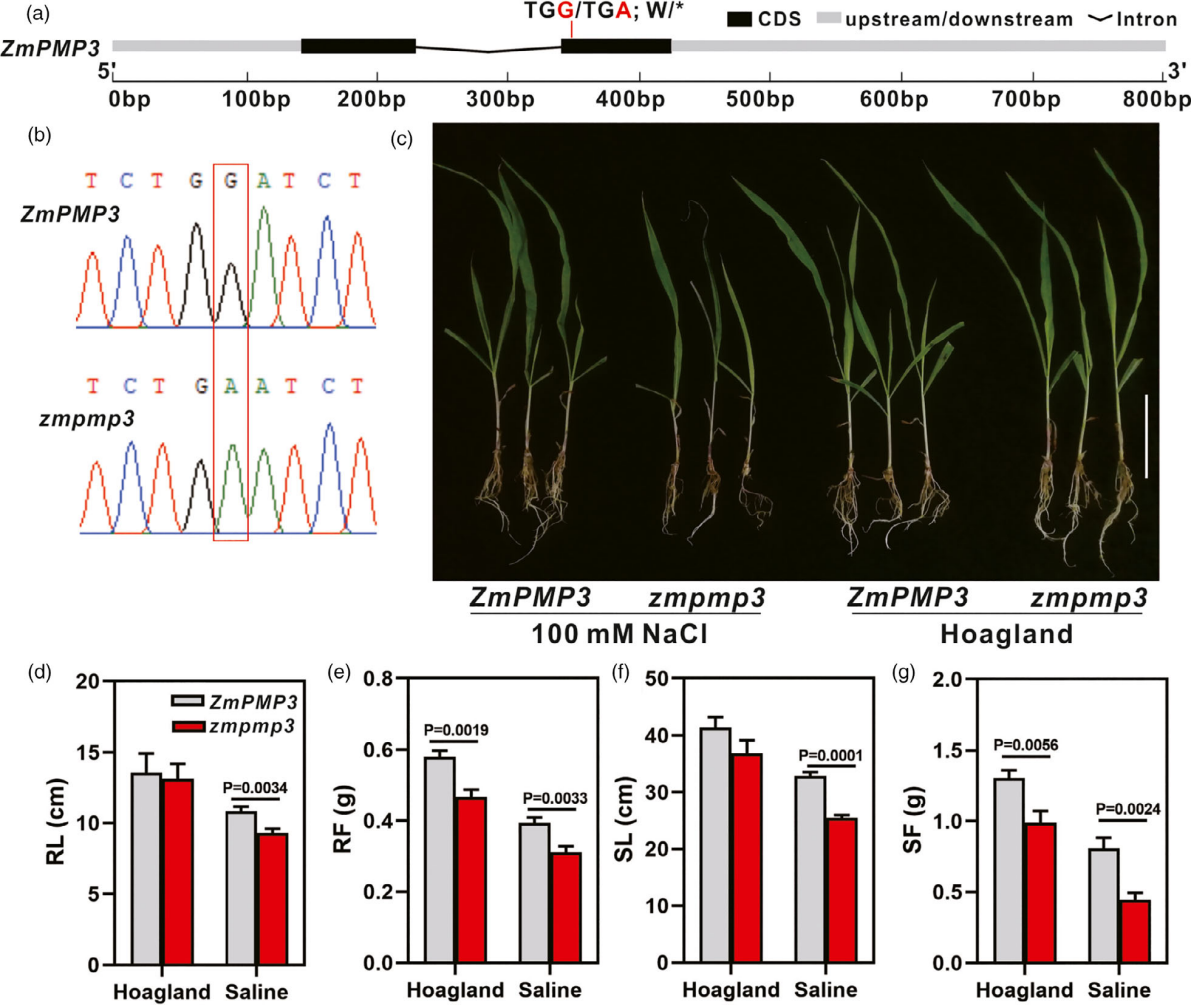
Growth parameters of *ZmPMP3* EMS mutant and the wild type (B73) under control and saline conditions. (a) Gene structure of *ZmPMP3* and the mutant site. (b) Sequencing chromatograms of *ZmPMP3* in wild type and the EMS mutant covering the mutation site. (c) Growth morphologies of the wild‐type B73 and the EMS mutant line after a 7‐day exposure to saline and control treatment. Bar = 10 cm. (d–g) Root length (RL) (d), root fresh weight (RF) (e), shoot length (SL) (f) and shoot fresh weight (SF) (g) of wild type and the EMS mutant maize line under control and saline conditions. Data are shown as the mean ± SD of three independent experiments. The *P* values were calculated by a two‐tailed Student’s *t* test.

To verify the salt tolerance function of candidate gene *ZmPMP3*, one B73 EMS mutant (Mut_Sample: EMS4‐0a0498) with termination mutation in the second exon of *ZmPMP3* was obtained from maize EMS mutant library (http://www.elabcaas.cn/memd/) (Figure 5a,b). The biomass of the *zmpmp3* mutant and its wild type was determined under control and salt treatment. We observed that the *zmpmp3* mutant showed significantly decreased root length, root fresh weight, shoot length and shoot fresh weight than the wild type under 100 mm NaCl condition (Figure 5c–g). These results indicated that *ZmPMP3* played a role in maize salt tolerance.

## Discussion

Maize is sensitive to salt stress, but is often planted on salt‐contaminated land because most farmland is salinized. Therefore, elucidating the genetic architecture of salt tolerance in maize is instrumental for improving its salt tolerance. In this study, a GWAS analysis based on SNP markers was used to dissect the genetic basis of salt‐related traits, including seedling length, fresh weight, dry weight and salt tolerance indexes. Multiple QTLs were identified for each trait, with some QTLs being simultaneously detected from several salt‐related traits. Moreover, candidate genes involved in salt tolerance were predicted and analysed, which may serve as potential targets in studies on the molecular mechanisms underlying salt tolerance in maize.

All of the traits were seriously inhibited under salt stress (Figure 1), consistent with previous reports (Luo *et al*., 2019a; Luo *et al*., 2017a; Luo *et al*., 2018). The pairwise correlations among nine growth parameters in both salt‐stressed and control conditions were significantly positive (*r* = 0.22–0.96), demonstrating strong genetic correlations among them. The salt tolerance indexes of some traits were weakly correlated with the growth parameters in control and salt stress conditions (Figure S2), indicating that different genetic mechanisms may control these traits.

In previous studies, GWAS analyses have successfully identified genomic regions associated with tolerance to various abiotic stresses (Zhang *et al*., 2013; Zhao *et al*., 2018). In the present study, the 2300‐Mb whole maize genome was covered by 557 894 high‐density SNPs, with an average interval of 4.1 kb between SNPs, allowing for fine‐resolution QTL mapping. Population structure is a main limitation in GWAS studies because it induces false‐positive associations. Several statistical methods were evaluated to reduce false positives (Lu *et al*., 2018; Zhang *et al*., 2016), and the results suggested that the Q + K mixed linear model had a greater ability to correct both false‐positive and false‐negative associations than the K and Q models. The QQ plot for SLS generated by three models we compared in this study (Figure S3). Consistent with previous studies (Lu *et al*., 2018; Zhang *et al*., 2016), results indicated that the Q as well as the Q + K model could better control the false‐negative errors than the K model, and the Q + K model was more reliable than the Q model. Therefore, we used K + Q model for GWAS analysis in this study.

Through the association mapping of root and shoot traits under control conditions, 21 QTLs were identified across all of the chromosomes, and 16 of the QTLs were related to root growth. Six QTLs associated with root growth matched to previously reported QTL regions (Burton *et al*., 2014; Cai *et al*., 2012; Li *et al*., 2015). Four QTLs controlling root length (located in 45.42–45.62‐Mb and 37.56–37.76‐Mb regions on chromosome 1, 171.53–171.73‐Mb region on chromosome 4, and 149.72–149.92‐Mb region on chromosome 6) co‐localized with *qSolPriLen1*, *qARL21‐1*, *qTRL14‐1* and *qARL26‐1*, respectively, which were identified from linkage populations in previous studies (Burton *et al*., 2014; Cai *et al*., 2012). Another QTL for RF, which was located at the 105.95–106.15‐Mb region on chromosome 10, co‐localized with *qRDW110‐1* for RD, which was identified from a BC_4_F_3_ maize population (Cai *et al*., 2012). A QTL localized in the 136.76–136.97 Mb region on chromosome 7 co‐localized with *qRDW7* for RD, which was identified from a recombinant inbred line population (Li *et al*., 2015). Two novel QTLs were detected by multiple traits (Table S4). Traits that shared the same QTLs were closely and significantly correlated with each other (*r* = 0.72–0.92), consistent with a previous study (Lu *et al*., 2018). Thus, the association analysis data appeared to be reliable. The novel QTLs for maize seedling growth identified in this study might enhance our understanding of the genetic basis for maize growth and development.

Only a few studies have tried to genetically map salt tolerance in maize (Cui *et al*., 2015; Luo *et al*., 2019b; Luo *et al*., 2019a; Luo *et al*., 2017b; Sandhu *et al*., 2020; Xie *et al*., 2019). The GWAS analysis in this study builds on the results of those studies and allowed us to identify salt tolerance‐related SNP markers throughout the genome. Based on 18 traits, 83 QTLs distributed across all 10 maize chromosomes were significantly associated with salt tolerance. The proportion of phenotypic variation explained by individual significant SNPs was less than 11.2%, implying that salt tolerance is a complex minor‐effect quantitative trait. Among the QTLs, 11 matched to those reported in previous studies (Cui *et al*., 2015; Luo *et al*., 2019b; Luo *et al*., 2019a; Luo *et al*., 2017b). For example, one QTL covering the region from 147.06 to 147.26 Mb on chromosome 1 was located within *qSPH1* (Luo *et al*., 2017b), *QStr1* (Cui *et al*., 2015), *QTwc1* (Cui *et al*., 2015) and *QSkcs|skcn1* (Cui *et al*., 2015), which were detected in linkage analyses, implying that our results were reliable. The significant SNPs of chr3.S_3201547 and chr3.S_3206938 for survival rate identified by Luo *et al*. (2019b) had strong LD with the significant SNP of chr3.S_3208836 for root length under salt stress identified in this study (*r^2^* > 0.2). In addition, 11 QTLs were identified by multiple traits (Table S4), suggesting that they had pleiotropic effects on salt tolerance. As expected, traits that shared the same QTLs had significant positive correlations with one another (*r* = 0.426–0.962).

Within the 83 QTL regions, 420 genes were identified (Table S5), and 367 genes were assigned to 24 Eukaryotic Orthologous Groups (KOG) categories. The main KOG classifications were “Signal transduction mechanisms” (30.2%), “Posttranslational modification, protein turnover, chaperones” (25.1%), “Transcription” (15.5%), “Carbohydrate transport and metabolism” (11.7%), and “Intracellular trafficking, secretion and vesicular transport” (11.4%) (Figure S7).

The most promising candidate genes were identified based on their functional annotations and their homologs as screening references. Finally, we identified 13 candidate genes located in 12 corresponding QTLs (Table 2). The phenotypic differences reached significant levels (*P* < 0.001 or 0.01) between the two alleles of each of the strongest trait‐associated SNPs (Figure S8). The *ZmPMP3* (*GRMZM2G477325*) was reported to regulate ion homeostasis in yeast mutant under salt stress (Fu *et al*., 2012). It was located within a QTL containing three leading SNPs (chr7.S_168404086, chr7.S_168404089 and chr7.S_168405013) on chromosome 7 (Figure S8b), and this QTL was simultaneously identified by five traits (Table S4). The biomass of the *zmpmp3* mutant decreased significantly under 100 mM NaCl condition compared to its wild type, which verified that *ZmPMP3* conferred maize salt tolerance (Figure 5). Another candidate gene, *GRMZM2G071119* (*ZmCLCg*) was located in a QTL (5.74–5.95 Mb, chromosome 2) harbouring 11 peak SNPs (Figure S8a). The *AT5G33280* encodes a voltage‐gated chloride channel responsible for chloride transmembrane transport in Arabidopsis (Nguyen *et al*., 2016), and *ZmCLCg* is homologous to *AT5G33280*, indicating that it is a promising target gene for salt tolerance. Maize *zmclcg* knock‐out mutants were generated using the CRISPR/Cas9 technology, and we found that the biomass of three *zmclcg* mutants were significantly lower than that of the wild type under salt stress, which confirmed the role of *ZmCLCg* in maize salt tolerance (Figure 4). Thus, these results verified the accuracy of our GWAS results and indicated that GWAS is an effective strategy to uncover DNA regions related to salt tolerance in maize.

Two genes encoding transcription factors, *myb73* (*GRMZM2G121570*) and *ereb137* (*GRMZM2G028386*), were located within two loci that explained more than 10% of phenotypic variation (Table 2). Moreover, these two regions were detected simultaneously by three traits (Table 2). The peak SNP of chr1.S_201957459 was located within *GRMZM2G028386*. Thus, these two transcription factors may play important roles in salt tolerance.

The hot spot on chromosome 5 contained four candidate genes: *GRMZM2G102754*, *GRMZM2G176085*, *GRMZM2G027351* and *GRMZM2G166049*. *GRMZM2G102754* is a homolog of Arabidopsis *ethylene insensitive 2*, which is involved in ethylene signal transduction and leaf senescence regulation under salt stress (Zhang *et al*., 2017). *GRMZM2G102754* harboured the peak SNP of chr5.S_12267893. *GRMZM2G176085* is a homolog of a peroxidase superfamily protein in Arabidopsis that responds to oxidative stress (Valério *et al*., 2004). Both *cdpk18* (*GRMZM2G027351*) and *mab17* (*GRMZM2G166049*) were located in the same QTL interval identified by two traits, and encode proteins that may be involved in signal transduction and transcriptional regulation under salt stress conditions (Mittal *et al*., 2017).

*bZIP108* (*GRMZM2G033230*) harboured the peak SNP of chr8.S_120609841, and its expression increased at all examined time points after salt treatment. Therefore, it is another likely candidate gene for salt tolerance. *vpp3* (*GRMZM2G421857*) encodes a vacuolar proton pump and showed significantly increased transcript levels in root from 2–48 h post salt treatment. *aasr1* (*GRMZM2G136910*) on chromosome 10 was adjacent to the peak SNPs chr10.S_8805555 and chr10.S_8848948, and encodes a protein that protects maize kernel yield under water deficiency conditions (Virlouvet *et al*., 2011). All these candidate genes may be involved in maize salt tolerance.

The transcript levels of 12 candidate genes fluctuated from 0.5–168 h after salt treatment and were up‐regulated at certain time points (Figure 3), indicating that all these genes were responsive to salt stress in maize. Consistent with the results of Fu *et al*. (2012), the expression of *ZmPMP3* showed a fluctuating trend, which was up‐regulated at some time points and down regulated at some time points (Fu *et al*., 2012).

Overall, 13 candidate genes which may be related to maize salt tolerance were identified by GWAS mapping in this study. Two candidate genes of *ZmCLCg* and *ZmPMP3* were selected for functional verification. The *zmclcg* mutants were obtained by target site editing with an efficient CRISPR/Cas9 vector carrying three tandemly arrayed tRNA‐target‐gRNA. Three *zmclcg* mutants were obtained, with one mutant had mutations in both exons 3 and exon 4. The biomass of three *zmclcg* mutants and one *zmpmp3* EMS mutant were compared with their wild‐type plants under 100 mm NaCl treatment, respectively, and the results showed that the root length, root fresh weight, shoot length, shoot fresh weight of all mutants were significantly lower than that of the wild type, which verified the salt tolerance function of both *ZmPMP3* and *ZmCLCg*. Chloride content analysis further indicated that *ZmCLCg* was associated with chloride transport in maize.

## Experimental procedures

### Plant materials and treatments

The association mapping panel consisted of 348 maize inbred lines, with 141 from tropical and subtropical zones and 126 from the temperate zone (Li *et al*., 2013; Liu *et al*., 2017; Yang *et al*., 2011b). The *ZmCLCg* knock‐out maize lines were obtained by CRISPR/Cas9 technology. The EMS mutant of *ZmPMP3* (Mut_Sample: EMS4‐0a0498) was obtained from the maize EMS mutant library (http://www.elabcaas.cn/memd/). The *ZmPMP3* gene (GenBank number: MW113229), *ZmCLCg* gene (GenBank number: MW113230) sequences of B73 maize inbred line, and the *ZmCLCg* gene sequence of B104 (GenBank number: MW113231) maize inbred line had been submitted to GenBank database. Primers for PCR amplification of full length of the *ZmCLCg* gene are listed in the Table S6.

Maize seeds were sterilized with 1% v/v NaClO for 10 min, and then rinsed three times with sterile water. Subsequently, sterilized maize seeds were sown and hydroponically cultured in a maize seedling identifying apparatus (Chinese patent number: ZL200920177285.0) placed in a greenhouse at 26 ± 1 °C and 60% relative humidity under a 12‐h light/12‐h dark (150–180 μmol m^−2^ s^−1^) photoperiod. Details of the apparatus’s operation are as follows: Maize seeds were fixed between two plates (170 mm × 50 mm), and the two plates were inserted into grooves on the inside surface of a container (325 mm × 190 mm × 95 mm). Each container had 10 grooves on the inside surface. Filter paper sheets were placed between the seeds and the plates and were humidified with 16‐mm deep nutrient solution (800 mL). All maize materials were laid out in a randomized complete‐block design with three replications (10 plants per replication).

For the association panel, the seedlings were hydroponically grown in sterile water (control) or sterile saline water containing 100 mm NaCl (salt treatment) for 10 days (Luo *et al*., 2019a; Yang *et al*., 2011a), and then the root and shoot traits were measured. For *ZmCLCg* CRISPR/Cas9 knock‐out maize lines and *ZmPMP3* EMS mutant, maize seeds were hydroponically grown in Hoagland’s nutrient solution [6 mm KNO_3_, 4 mm Ca(NO_3_)_2_·4H_2_O, 1 mm NH_4_H_2_PO_4_, 0.047 mm ethylenediamine tetraacetic acid, disodium ferric salt, 2 mm MgSO_4_·7H_2_O, 0.0095 MnSO_4_·4H_2_O, 0.7 μm ZnSO_4_·7H_2_O, 0.046 mm H_3_BO_3_, 0.3 μm CuSO_4_·5H_2_O and 0.016 μm ammonium molybdate tetrahydrate] for 11 days, and then were cultured in Hoagland’s nutrient solution (control) or in Hoagland’s nutrient solution containing 100 mm NaCl (salt treatment) for 7 days. The culture solution was replaced with fresh solution on day (d) 4 of culture, and every 2 d after that.

For the gene expression profile analysis, seeds of maize inbred line Jing724 or B73 were hydroponically cultured for 11 d. During this period, maize seedlings were hydroponically cultured in sterile water for the first 3 d, and then in Hoagland’s nutrient solution for another 8 d (Luo *et al*., 2018). On d 12, the culture solutions were replaced with Hoagland’s nutrient solution containing 100 mm NaCl (salt treatment). After salt treatment for 0, 0.5, 1, 2, 4, 8, 12, 24, 48, 72, 120 and 168 h, seedlings of Jing724 were harvested for transcriptome analyses. After salt treatment for 0, 1, 2, 4, 8, 12, 24, 48 and 72 h, shoots of B73 seedlings were harvested for quantitative real‐time reverse‐transcription PCR (qRT‐PCR) analyses. Three biological replicates were analysed.

### Phenotypic data collection

The SLs and RLs of maize seedlings were measured with a ruler. The fresh and dry weights of seedling shoots (SF and SD, respectively) and roots (RF and RD, respectively) were measured using an electronic analytical balance. For dry weight determination, fresh shoot and root samples were oven‐dried at 80°C for 3 d and then weighed. Ten seedlings for each replicate and three biological replicates were analysed. After measurements, the salt tolerance index for each trait was calculated using the formula: salt tolerance index for each trait = measured value under salt stress/ measured value under normal condition. Phenotypic trait distributions, correlations and frequency distributions were determined using R version 3.4.4 (http://www.r‐project.org/).

The chloride content was determined by ion chromatography. Shoot of seedlings were dried, grounded and was passed through 40‐mesh sieve. Transfer 0.2 g sample into a 50 ml tube and add 10 ml solution containing 3.5 mm Na_2_CO_3_ and 1.0 mm NaHCO_3_. After digesting at 80 °C for 1 h, the digested solution was filtered through 0.45 μm membrane. Finally, the chloride content was determined using a Dionex ICS 600 ion chromatography (Thermo Fisher Scientific, Agawam, MA, USA) with a Dionex IonPac^TM^ AS14 chromatographic column (Thermo Fisher Scientific). The solution containing 3.5 mm Na_2_CO_3_ and 1.0 mm NaHCO_3_ was used as eluent and the flow rate was 1.0 ml/min.

### Genome‐wide association analysis

More than 1.06 million high‐quality SNPs obtained from an RNA‐seq project (Fu *et al*., 2013) combined with the Illumina MaizeSNP50 BeadChip (Ganal *et al*., 2011) were used for the GWAS analysis. The SNP data can be downloaded from http://www.maizego.org/Resources.html. The mean values of the three biological replicates for each trait of the maize association population under control and salt stress conditions were used as the inputs for the GWAS. The GWAS analysis and statistical model comparison were implemented in TASSEL 5.2.44 software (https://tassel.bitbucket.io/). A *P* value of 1.79E‐06 (1/number of markers with a minor allele frequency of ≥5%) was used to determine significant associations. Manhattan and QQ plots were constructed using R version 3.4.4.

### Candidate genes analysis

The reported maize B73 working gene list from the MaizeGDB database (http://www.maizegdb.org, RefGen_v2) was used to identify genes within each QTL. Genes were annotated according to the UniProtKB (https://www.uniprot.org/) and TAIR (https://www.arabidopsis.org/) databases. According to the LD of the association population, all genes and their annotations within 200 kb (100 kb up‐ and downstream) of significant loci were identified (Li *et al*., 2013; Liu *et al*., 2017; Wang *et al*., 2019). For functional classifications, all genes were used as queries in searches against the KOG (https://www.ncbi.nlm.nih.gov/COG/) database. For candidate gene analysis, all genes were used as queries in searches against the MaizeGDB, Gramene (http://www.gramene.org/) and TAIR databases, and their functional annotations were confirmed. If the annotations showed that they were related to salt stress or water deficiency, then they were considered as candidate genes for salt tolerance.

### Candidate gene expression profiles

The expression profiles of candidate genes were determined in a transcriptome analysis. The transcriptome library preparation and sequencing were performed by Annoroad Gene Technology Co., Ltd. (Beijing, China) (Yu *et al*., 2017). Total RNA was extracted from shoots and roots of maize seedlings using TRIZOL (Invitrogen, Carlsbad, CA), and the purity, integrity and concentration were checked by electrophoresis and the Bioanalyzer 2100 system (Agilent Technologies, Santa Clara, CA). The transcriptome libraries were generated using the NEBNext® Ultra™ II Directional RNA Library Prep Kit for Illumina® (New England Biolabs, Ipswich, MA) according to manufacturer’s instructions. The quality and quantity of the libraries were controlled with the Bioanalyzer 2100 system (Agilent Technologies) and a CFX96 Real‐Time PCR Detection System (Bio‐Rad, Hercules, CA). The resulting libraries were sequenced using a HiSeq X ten instrument (Illumina, San Diego, CA). The raw sequences were cleaned by removing low‐quality reads and reads containing adaptors. The cleaned reads were mapped to the maize reference genome (www.maizegdb.org) to obtain expression data for the identified genes. The sequencing data had been deposited in the Sequence Read Archive database (Accession number: PRJNA670840).

For qRT‐PCR analysis, total RNA was extracted using TRIZOL reagent (Invitrogen, Carlsbad, CA), and then was reverse‐transcribed using a PrimeScript™ II 1st strand cDNA Synthesis Kit (Takara Bio Inc., Shiga, Japan). qRT‐PCR was performed using SYBR Premix Ex TaqII (Takara Bio Inc., Shiga, Japan). The amplification was carried out in a QuantStudio™ 6 Flex (ABI Life Technologies, Carlsbad, CA, USA) system. The *ZmActin1* was used as the internal reference. The mRNA relative expression levels were analysed using the 2^−▵▵Ct^ method (Luo *et al*., 2017b). Primers for qRT‐PCR were listed in the Table S6.

### Generation of *ZmCLCg* CRISPR/Cas9 knock‐out maize lines

The pCambia2300‐Spe vector containing the SpCas9n‐pBE construct was used for plasmid construction (Wu *et al*., 2019). In the CRISPR/Cas9 vector, the sequences of U3 promoter, tRNA, esgRNA and the protein sequence of SpCas9 are according to previous reports (Cong *et al*., 2013; Xie *et al*., 2015). In addition, the coding sequence of SpCas9 was codon‐optimized for high expression in maize and was synthesized by Nanjing Kingsley Biotechnology Co., Ltd. For CRISPR/Cas9 vector construction, the Cas9n‐PmCDA1‐UGI‐t35s fragment in the SpCas9n‐pBE construct was replaced with Cas9‐t35s fragment using *Sna*BI and *Asc*I, and the ZmUbi1‐Hpt‐t35s fragment was replaced with the ZmUbi1‐bar‐t35s fragment using *Asc*I and *Avr*II. The CRISPR/Cas9 vector also contains a synthetic polycistronic gene that harbours three tandemly arrayed tRNA‐target‐gRNA (Xie *et al*., 2015). The three target sites located at the first, third and fourth exons of *ZmCLCg* were assembled with gRNA using *Bsa*I. The resulting vector was transformed into the *Agrobacterium* *tumefaciens* strain EHA105 (Weidi Biotech, Shanghai, China). The *Agrobacterium*‐mediated method was applied to transform immature embryos of maize inbred line B104. A total of 10 independent T0 transgenic plants were generated. The genomic fragments encompassing the target sites for each transgenic plant were sequenced by Sanger sequencing. No T0 plants with mutation at the target site of exon 1 were detected. One and seven T0 plant with mutation at the target site of exon 3 (Mutation efficiency = 10%) and exon 4 (Mutation efficiency = 70%) were identified, respectively. All T0 plants with mutation at the target sites were self‐pollinated to get T1 progenies. PCR products covering the target sites of all T1 plants were sequenced to identify T1 plants with homozygous mutation in target sites. The confirmed homozygous mutant T1 plants and the wild‐type plants were used for salt stress treatment and phenotypic determination. The primers for PCR amplification of target sites are listed in the Table S6.

### Statistical analyses

Comparisons of phenotypic data and gene expression levels were conducted by unpaired two‐tailed Student’s *t* tests. Student’s *t* tests and correlation analyses were conducted using the *t* test and correlation functions in GraphPad Prism 5 software (http://www.graphpad.com/), respectively. The means, standard deviations, coefficients of variation, kurtosis and skewness were calculated using the column statistics function in GraphPad Prism 5 software. Repeatability for each trait was determined using the R software (Luo *et al*., 2019). Repeatability was calculated with the formula: repeatability = $\sigma_{G}^{2}/\left(\sigma_{G}^{2}+\sigma_{e}^{2}\right)$, while $\sigma_{G}^{2}$ and $\sigma_{e}^{2}$ represent the genetic variance and the error variance, respectively.

## Conflict of interest

The authors declare no conflict of interest.

## Author contributions

Z.Y. (Yanxin Zhao), S.W. and Z.J. designed the experiment, conceived the project and supervised the study; L.M. conducted all the data analysis and wrote the manuscript; L.M., Z.Y. (Yunxia Zhang), L.J., Z.P. and C.K. performed the phenotyping; Y.J. performed the CRISPR/Cas9 editing; W.X., L.X. and L.B. analysed data. All authors reviewed the manuscript.

## Supporting information

**Figure S1** Frequency distributions of all 27 traits collected from the maize association panel. SL: shoot length, RL: root length, FL: full length of seedling, SF: shoot fresh weight, RF: root fresh weight, FF: full fresh weight of seedling, SD: shoot dry weight, RD: root dry weight, FD: full dry weight of seedling. SL, RL, FL, SF, RF, FF, SD, RD and FD represent traits under normal conditions; SLS, RLS, FLS, SFS, RFS, FFS, SDS, RDS and FDS represent traits under salt stress condition; SLR, RLR, FLR, SFR, RFR, FFR, SDR, RDR and FDR represent salt tolerance indexes of traits.
**Figure S2** Pearson’s correlation coefficients (*r*) between 27 maize traits (nine each in the control and under salt stress conditions, and nine salt tolerance indexes). Correlation coefficients were calculated from mean values of three biological replicates for each trait of the maize association population. See Figure S1 caption for abbreviations.
**Figure S3** Quantile–quantile (QQ) plots of genome‐wide association study (GWAS) results using different association models for maize shoot length trait under salt treatment conditions. Horizontal dashed red line represents significance threshold (1.79 × 10^–6^).
**Figure S4** Manhattan plots for all 27 maize traits using Q + K mixed linear model. See Figure S1 caption for abbreviations.
**Figure S5** QQ plots for all 27 maize traits using Q + K mixed linear model. See Figure S1 caption for abbreviations
**Figure S6** Growth status and growth parameters of B104, B73, the significantly salt‐tolerant maize inbred line GEMS37, and the significantly salt sensitive maize inbred line CIMBL157 after culturing in control or saline water for 10 days. (a) Growth status; (b) Root length; (c) Roof fresh weight; (d) Shoot length; (e) Shoot fresh weight. Data are shown as the mean ± SE of three independent experiments. The *P* values were calculated by a two‐tailed Student’s *t* test.
**Figure S7** KOG functional categories for maize genes within significant QTL regions
**Figure S8** Candidate regions associated with salt tolerance and phenotypic differences between two alleles of most significant trait‐associated single nucleotide polymorphisms (SNPs). SNPs within candidate regions are highlighted in green. Peak SNPs are marked by arrows. (a–d), Candidate regions associated with RLS, SFS, RLS and RLS located on chromosome 2, 7, 7 and 10, respectively (left). Phenotypic differences in RLS, SFS, RLS and RLS between two alleles of peak SNPs (right). ***P < 0.001

**Table S1** List of 348 maize lines used in this study

**Table S2** List of significant maize SNP‐trait associations and detailed information identified by GWAS

**Table S3** Numbers of significant loci for measured maize traits
**Table S4** Summary of significant loci identified by multiple maize traits

**Table S5** List of all genes within significant maize loci and their positional and annotational information
**Table S6** Primers used in this study
