# Supplementary material for: Molecular dissection of maize seedling salt tolerance using a genome‐wide association analysis method
Source: Plant Biotechnol J. 2021 May 2;19(10):1937–51. doi: 10.1111/pbi.13607 (PMC8486251; doi:10.1111/pbi.13607)
Supplement: Supplementary file 1 — Figure S1 Frequency distributions of all 27 traits collected from the maize association panel. SL: shoot length, RL: root length, FL: full length of seedling, SF: shoot fresh weight, RF: root fresh weight, FF: full fresh weight of seedling, SD: shoot dry weight, RD: root dry weight, FD: full dry weight of seedling. SL, RL, FL, SF, RF, FF, SD, RD and FD represent traits under normal conditions; SLS, RLS, FLS, SFS, RFS, FFS, SDS, RDS and FDS represent traits under salt stress condition; SLR, RLR, FLR, SFR, RFR, FFR, SDR, RDR and FDR represent salt tolerance indexes of traits. Figure S2 Pearson’s correlation coefficients (r) between 27 maize traits (nine each in the control and under salt stress conditions, and nine salt tolerance indexes). Correlation coefficients were calculated from mean values of three biological replicates for each trait of the maize association population. See Figure S1 caption for abbreviations. Figure S3 Quantile–quantile (QQ) plots of genome‐wide association study (GWAS) results using different association models for maize shoot length trait under salt treatment conditions. Horizontal dashed red line represents significance threshold (1.79 × 10^–6^). Figure S4 Manhattan plots for all 27 maize traits using Q + K mixed linear model. See Figure S1 caption for abbreviations. Figure S5 QQ plots for all 27 maize traits using Q + K mixed linear model. See Figure S1 caption for abbreviations Figure S6 Growth status and growth parameters of B104, B73, the significantly salt‐tolerant maize inbred line GEMS37, and the significantly salt sensitive maize inbred line CIMBL157 after culturing in control or saline water for 10 days. (a) Growth status; (b) Root length; (c) Roof fresh weight; (d) Shoot length; (e) Shoot fresh weight. Data are shown as the mean ± SE of three independent experiments. The P values were calculated by a two‐tailed Student’s t test. Figure S7 KOG functional categories for maize genes within significant QTL regions Figure S8 Candidate r [file PBI-19-1937-s003.pdf]

Supporting figures

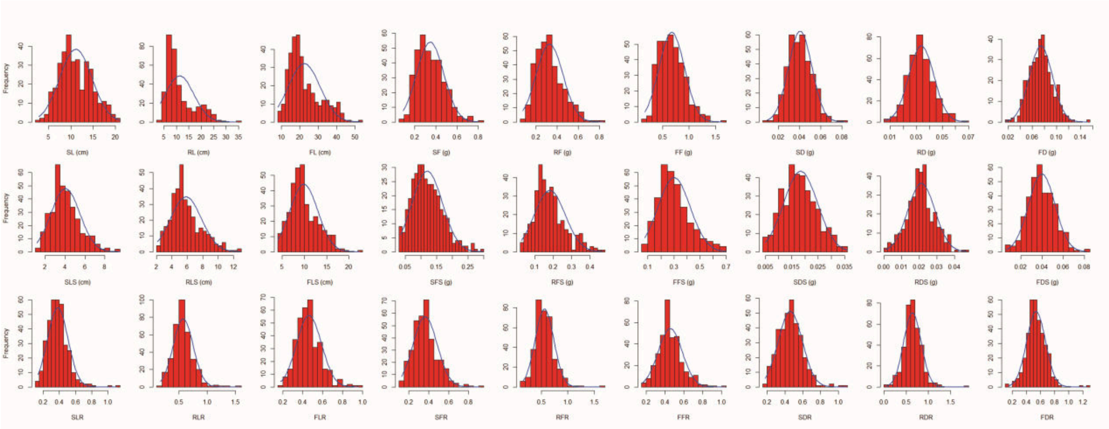

Figure S1. Frequency distributions of all 27 traits collected from the maize association panel. SL: shoot length, RL: root length, FL: full length of seedling, SF: shoot fresh weight, RF: root fresh weight, FF: full fresh weight of seedling, SD: shoot dry weight, RD: root dry weight, FD: full dry weight of seedling. SL, RL, FL, SF, RF, FF, SD, RD and FD represent traits under normal conditions; SLS, RLS, FLS, SFS, RFS, FFS, SDS, RDS and FDS represent traits under salt-stress condition; SLR, RLR, FLR, SFR, RFR, FFR, SDR, RDR and FDR represent salt tolerance indices of traits.

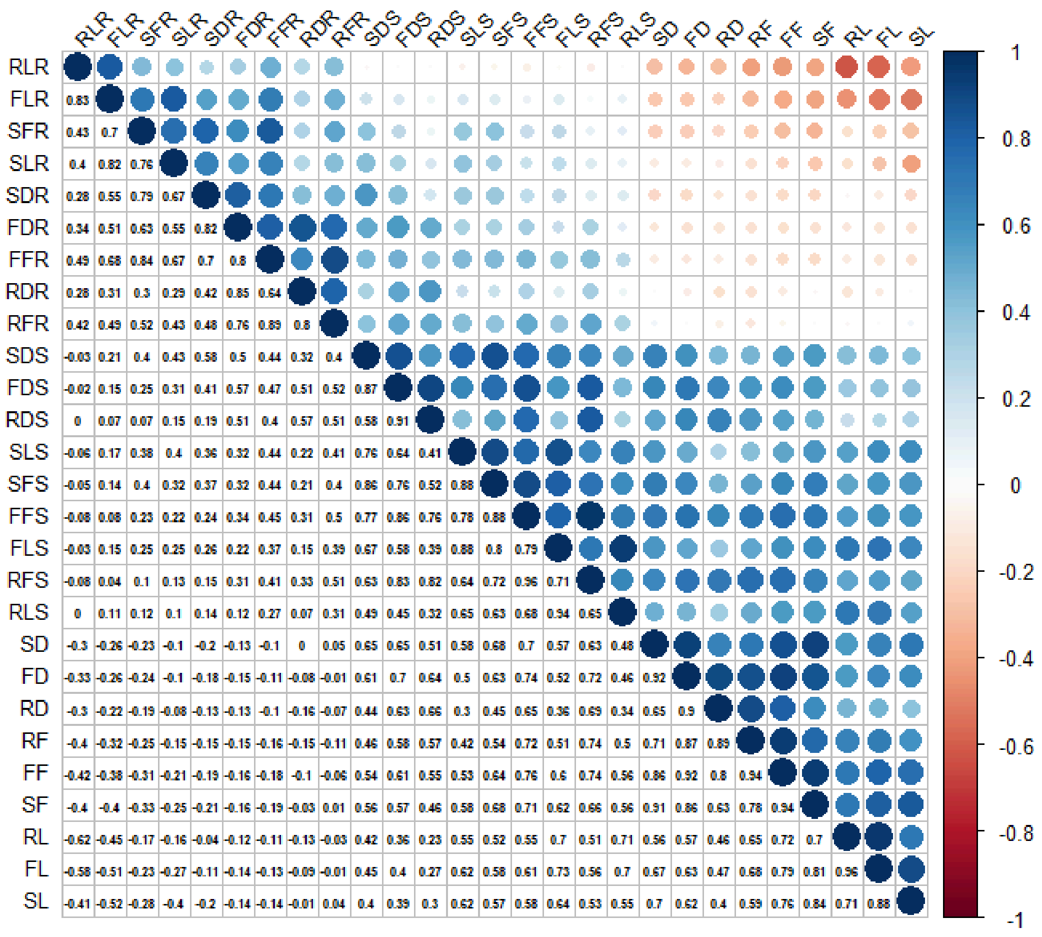

Figure S2. Pearson's correlation coefficients (*r*) between 27 maize traits (nine each in the control and under salt-stress conditions, and nine salt tolerance indexes). Correlation coefficients were calculated from mean values of three biological replicates for each trait of the maize association population. See Fig. S1 caption for abbreviations.

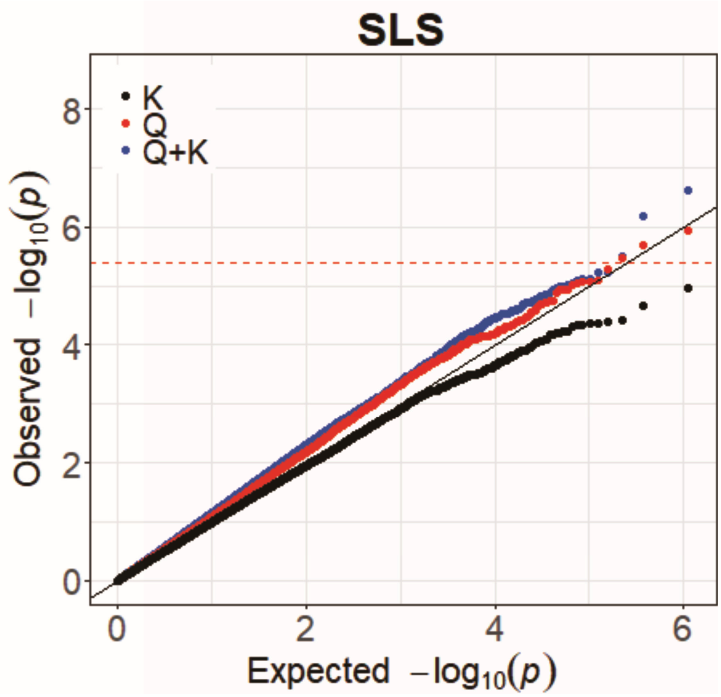

Figure S3. Quantile–quantile plots of genome-wide association study (GWAS) results using different association models for maize shoot length trait under salt-treatment conditions. Horizontal dashed red line represents significance threshold (1.79×10^{-6}).

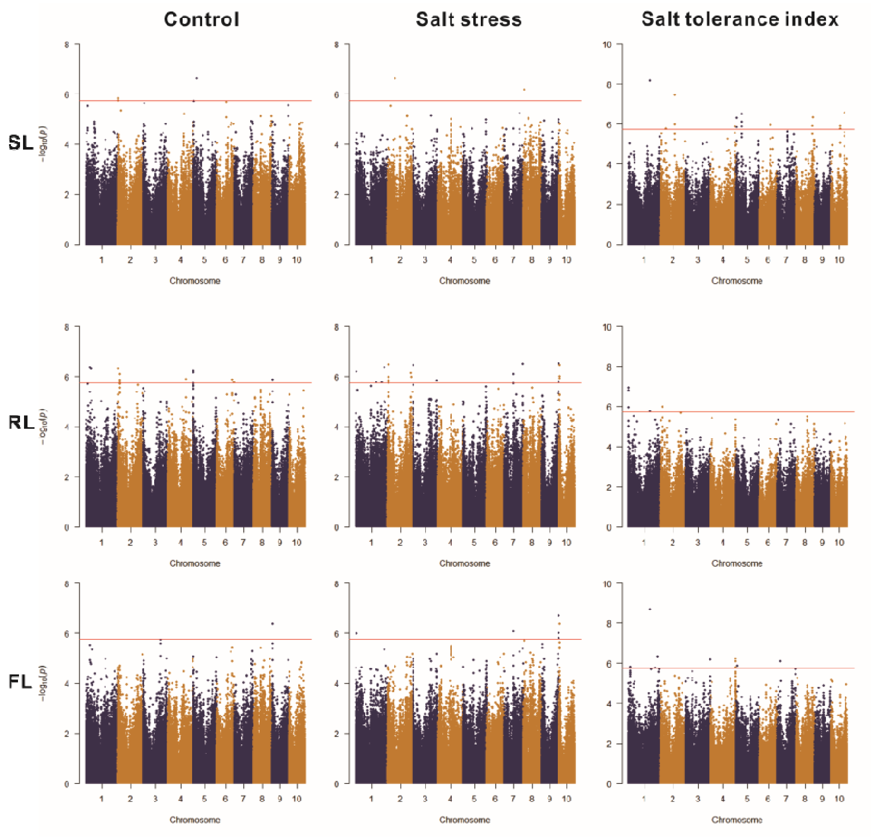
Control
Salt stress
Salt tolerance index
SL
RL
FL
$-\log_{10}(p)$
Chromosome

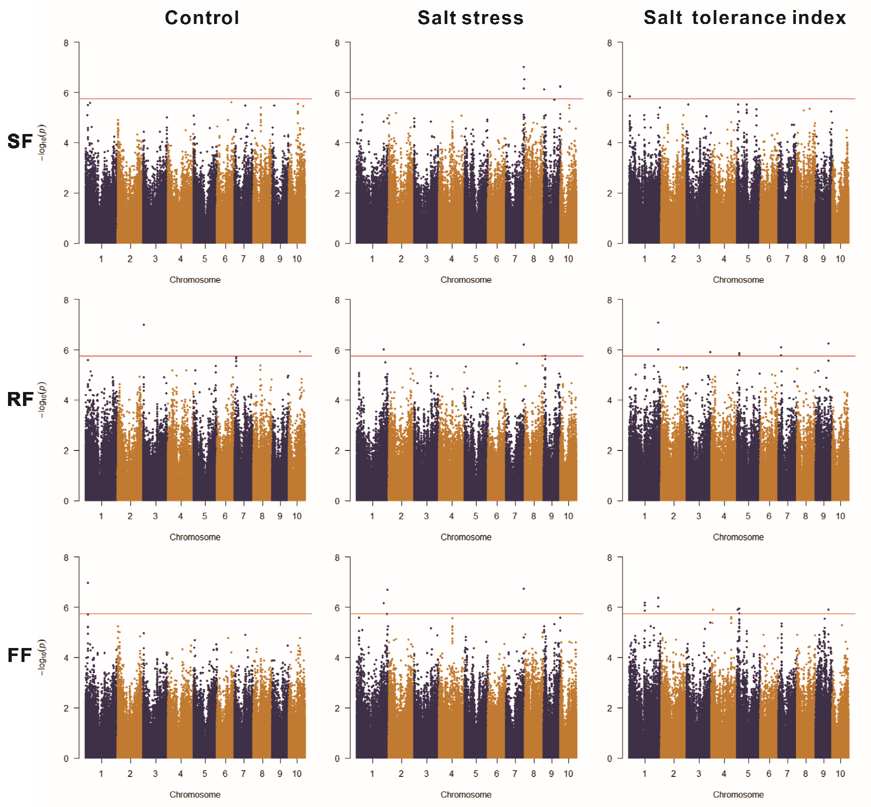
Control
Salt stress
Salt tolerance index
SF
RF
FF
$-\log_{10}(p)$
Chromosome

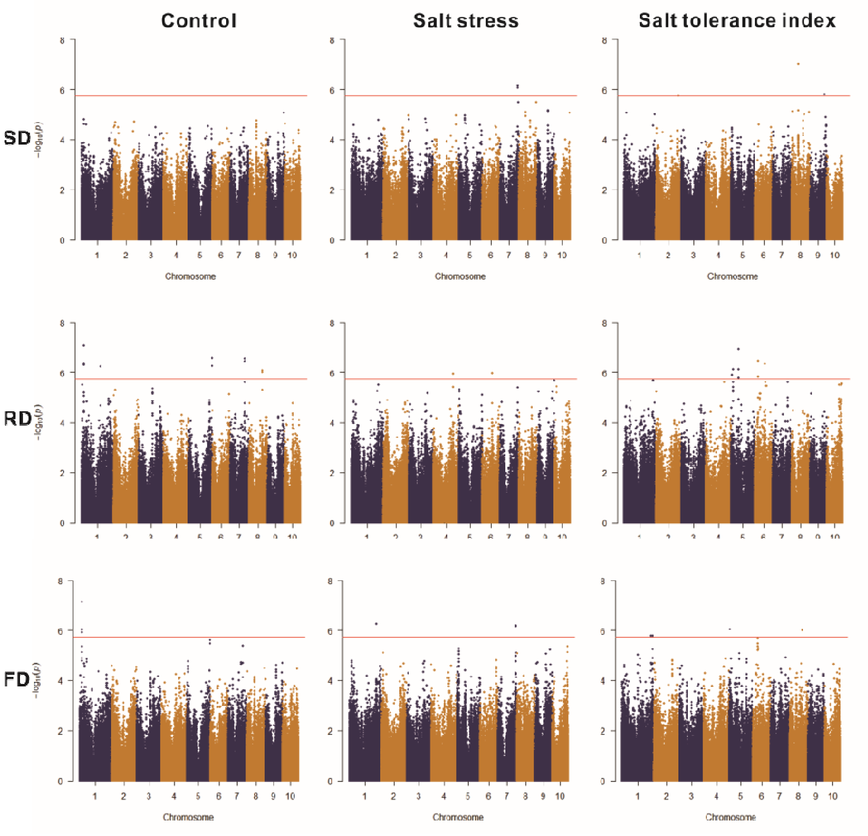

Figure S4. Manhattan plots for all 27 maize traits using Q+K mixed linear model. See Fig. S1 caption for abbreviations.

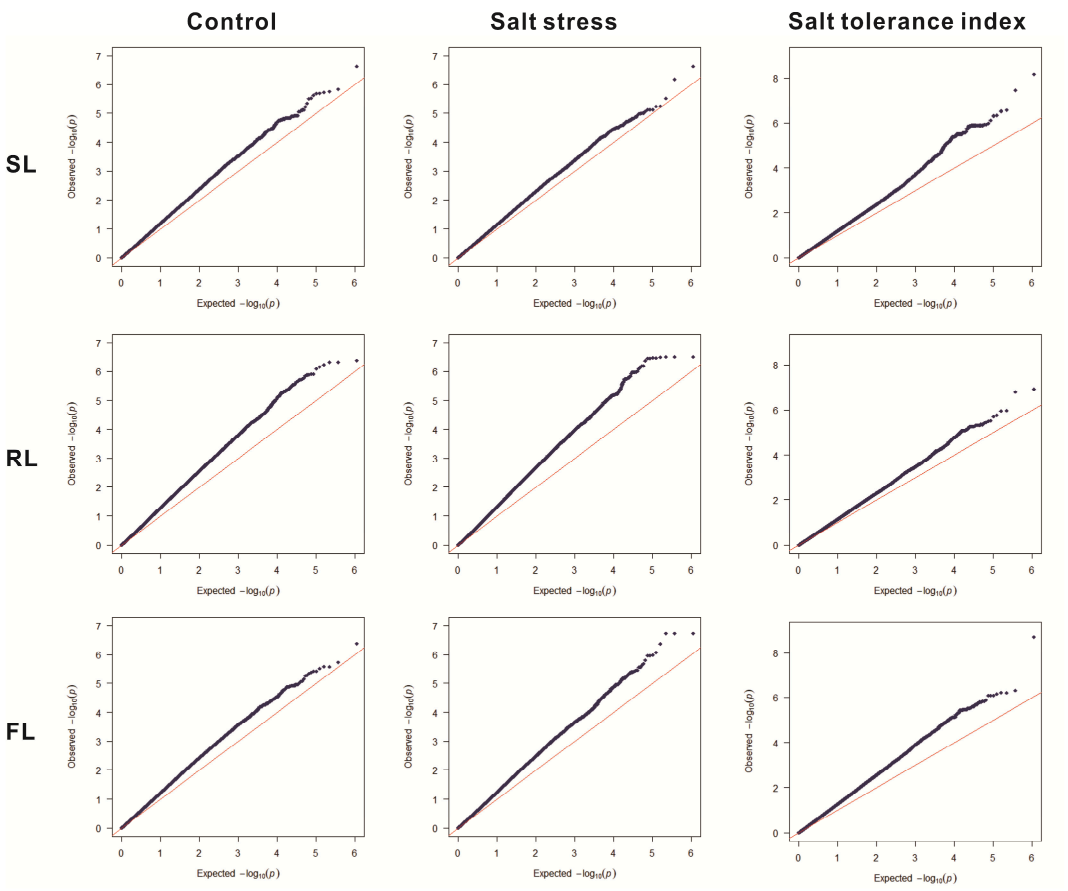
Control
Salt stress
Salt tolerance index
SL
RL
FL
Observed −log10(p)
Expected −log10(p)

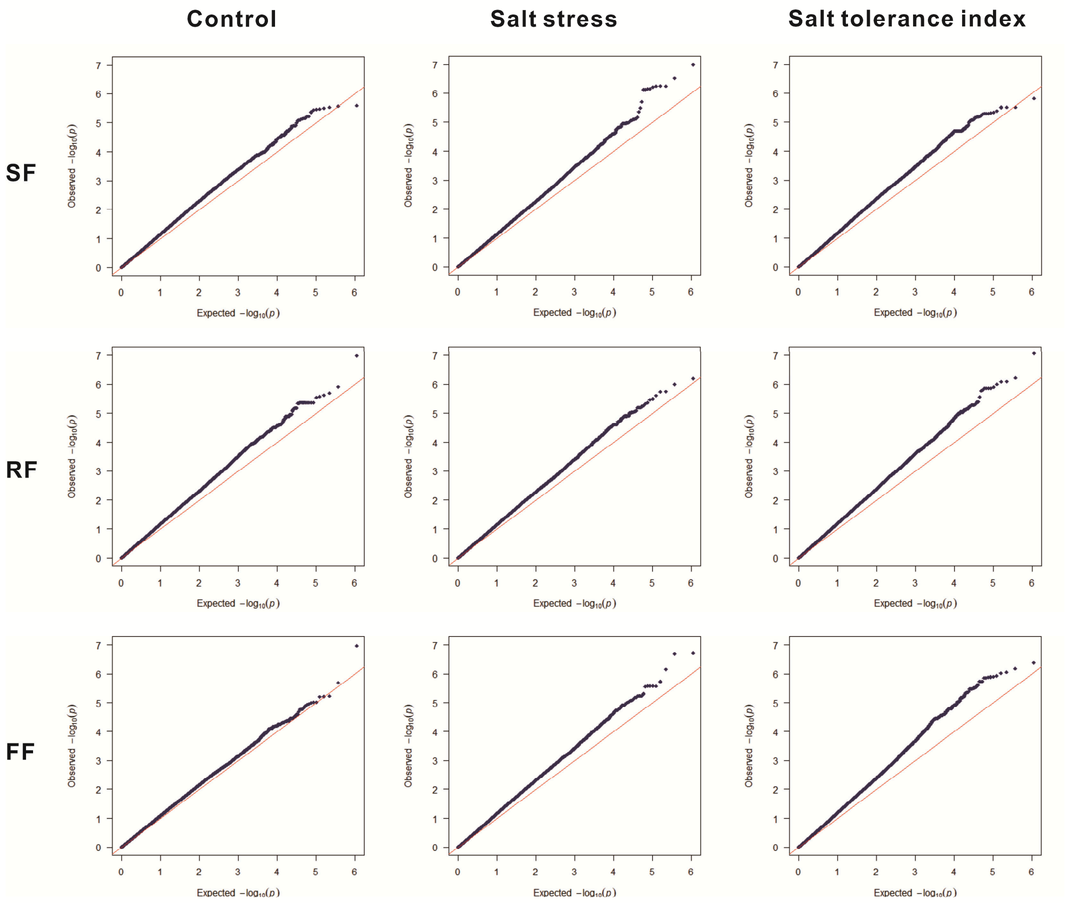
Control
Salt stress
Salt tolerance index
SF
RF
FF
Observed −log10(p)
Expected −log10(p)

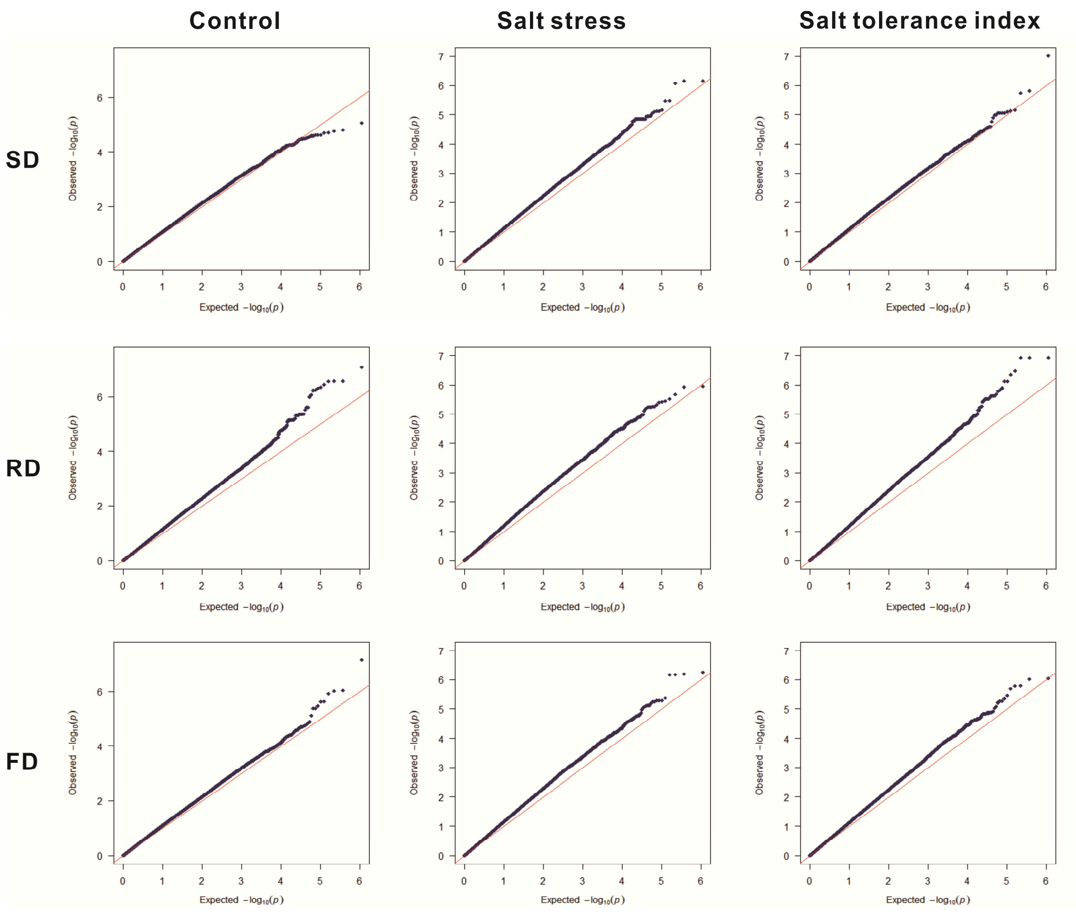

Figure S5. QQ plots for all 27 maize traits using Q+K mixed linear model. See Fig. S1 caption for abbreviations.

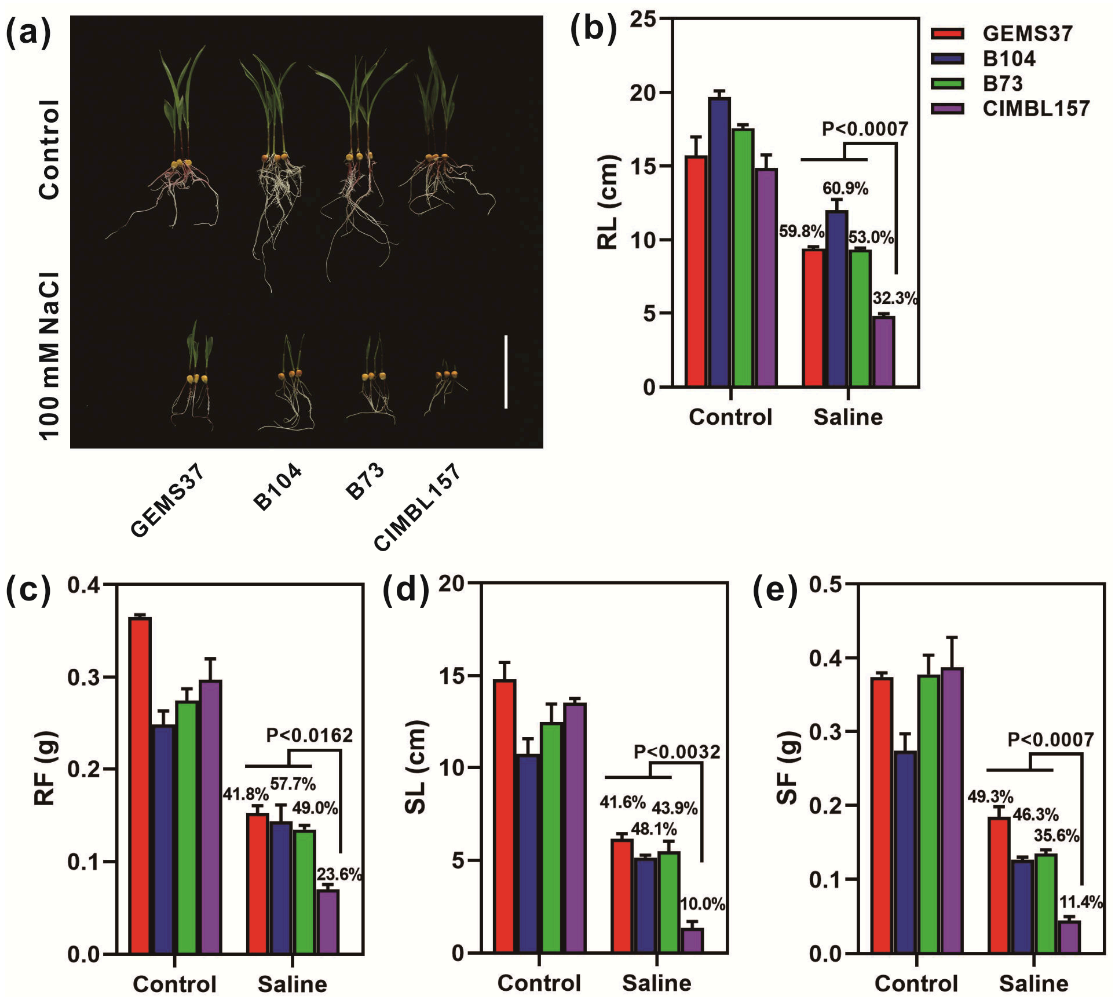

Figure S6. Growth status and growth parameters of B104, B73, the significantly salt tolerant maize inbred line GEMS37, and the significantly salt sensitive maize inbred line CIMBL157 after culturing in control or saline water for 10 days. (a) Growth status. Bar = 10 cm; (b) Root length; (c) Roof fresh weight; (d) Shoot length; (e) Shoot fresh weight. Data are shown as the mean ± SE of three independent experiments. The *P* values were calculated by a two-tailed Student's *t* test.

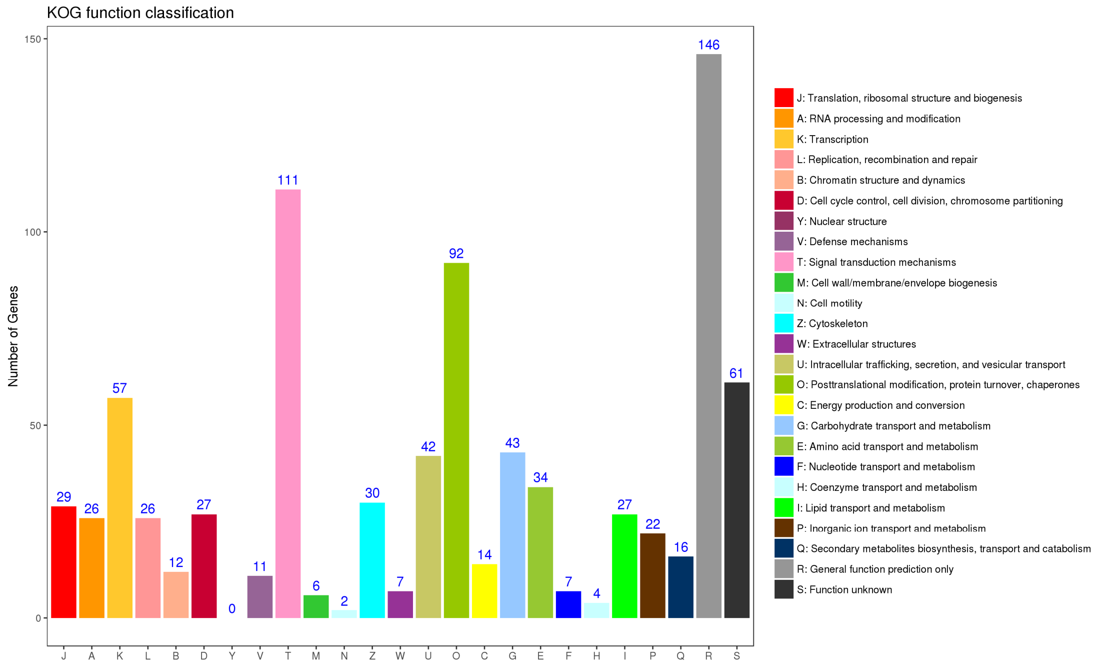

Figure S7. KOG functional categories for maize genes within significant QTL regions.

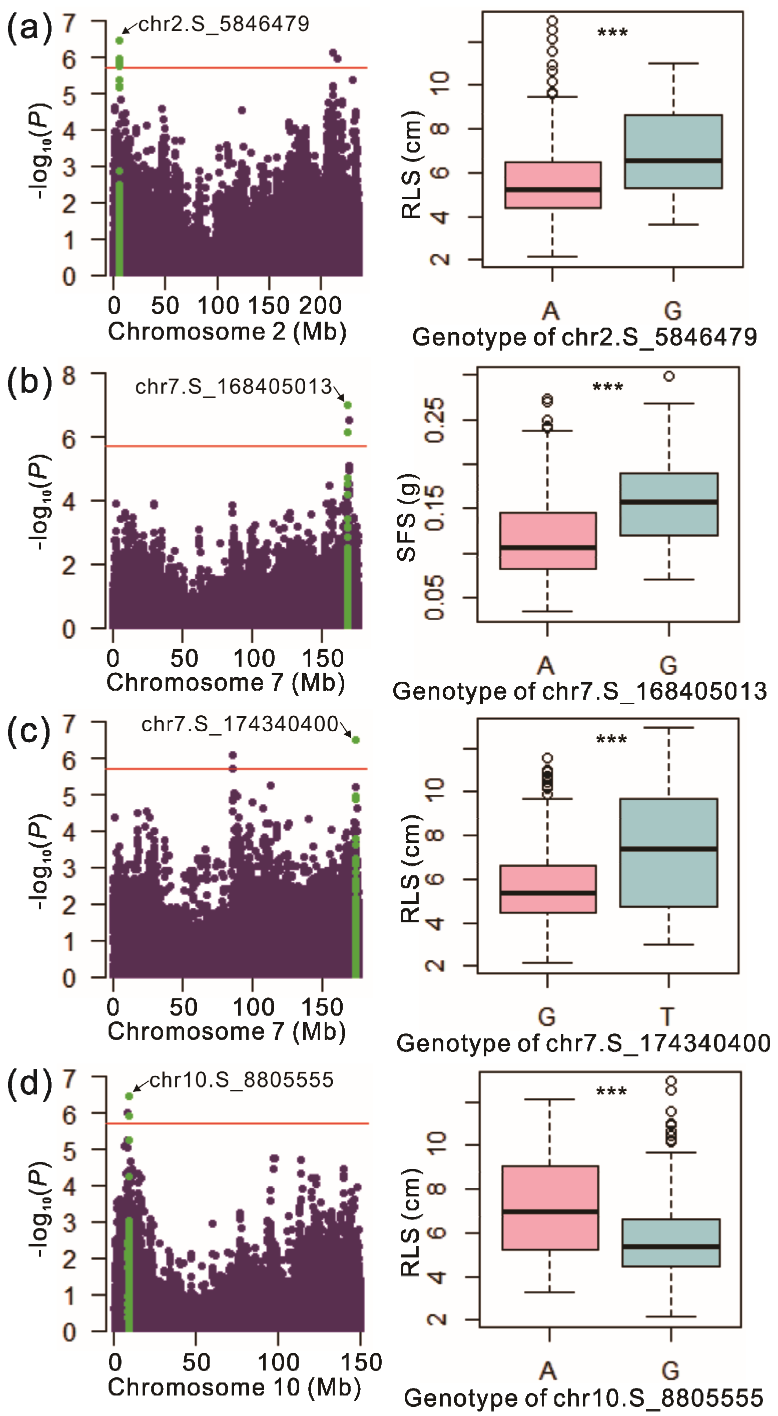

Figure S8. Candidate regions associated with salt tolerance and phenotypic differences between two alleles of most significant trait-associated single nucleotide polymorphisms (SNPs). SNPs within candidate regions are highlighted in green. Peak SNPs are marked by arrows. a–d, Candidate regions associated with RLS, SFS, RLS, and RLS located on chromosome 2, 7, 7, and 10, respectively (left). Phenotypic differences in RLS, SFS, RLS, and RLS between two alleles of peak SNPs (right). *** $P < 0.001$.
