# Supplementary material for: Molecular dissection of maize seedling salt tolerance using a genome‐wide association analysis method
Source: Plant Biotechnol J. 2021 May 2;19(10):1937–51. doi: 10.1111/pbi.13607 (PMC8486251; doi:10.1111/pbi.13607)
Supplement: Supplementary file 4 — Table S3 Numbers of significant loci for measured maize traits Table S4 Summary of significant loci identified by multiple maize traits [file PBI-19-1937-s002.docx]

**Supporting tables**

**Table S1.** List of 348 maize lines used in this study. (see Excel Table S1 file)

**Table S2.** List of significant maize SNP-trait associations and detailed information identified by GWAS. (see Excel Table S2 file)

**Table S3.** Numbers of significant loci for measured maize traits.

| **Traits** | **Numbers of significant loci under different conditions** | | |
| --- | --- | --- | --- |
|  | **Control** | **Salt stress** | **Salt tolerance index** |
| SL | 2 | 2 | 11 |
| SF | 0 | 5 | 1 |
| SD | 0 | 1 | 2 |
| RL | 9 | 14 | 4 |
| RF | 2 | 2 | 5 |
| RD | 5 | 2 | 5 |
| FL | 1 | 5 | 9 |
| FF | 1 | 3 | 6 |
| FD | 1 | 2 | 4 |

SL: shoot length, RL: root length, FL: full length of seedling, SF: shoot fresh weight, RF: root fresh weight, FF: full fresh weight of seedling, SD: shoot dry weight, RD: root dry weight, FD: full dry weight of seedling.

**Table S4.** Summary of significant loci identified by multiple maize traits.

| **Treatment** | **Traits** | **Chr.** | **Position (bp)** | ***P* value** |
| --- | --- | --- | --- | --- |
| Salt stress | RLS, FLS | 1 | 7,358,549 - 7,558,550 | 6.5547E-07 |
|  | RLR, SLR, FLR | 1 | 201,857,459 - 202,057,459 | 2.0634E-09 |
|  | RFS, FDS, FFS | 1 | 252,259,061 - 252,459,061 | 5.6507E-07 |
|  | RFR, FFR, FLR | 1 | 275,733,344 - 275,936,214 | 8.7404E-08 |
|  | RDR, FDR | 5 | 4,565,179 - 4,765,179 | 9.0689E-07 |
|  | RFR, FFR | 5 | 19,145,023 - 19,346,728 | 1.1565E-06 |
|  | RLS, FLS | 7 | 86,096,089 - 86,296,089 | 8.1246E-07 |
|  | SDS, SFS, FDS, RFS, FFS | 7 | 168,304,086 - 168,505,013 | 9.9881E-08 |
|  | SFS, FLS | 9 | 154,236,524 - 154,436,524 | 6.2204E-07 |
|  | RLS, FLS, SFS | 9 | 154,681,818 - 154,885,711 | 1.9403E-07 |
|  | RLS, FLS | 10 | 8,351,541 - 8,551,541 | 4.3519E-07 |
| Control | RD, FD, FF | 1 | 21,658,969 - 21,859,614 | 7.05E-08 |
|  | RL, FL | 9 | 7,624,127 - 7,824,127 | 4.25E-07 |

SL: shoot length, RL: root length, FL: full length of seedling, SF: shoot fresh weight, RF: root fresh weight, FF: full fresh weight of seedling, SD: shoot dry weight, RD: root dry weight, FD: full dry weight of seedling. SL, RL, FL, SF, RF, FF, SD, RD and FD represent traits under normal conditions; SLS, RLS, FLS, SFS, RFS, FFS, SDS, RDS and FDS represent traits under salt-stress condition; SLR, RLR, FLR, SFR, RFR, FFR, SDR, RDR and FDR represent salt tolerance indexes of traits.

**Table S5.** List of all genes within significant maize loci and their positional and annotational information. (see Excel Table S5 file)

**Table S6.** Primers used in this study.

|  | **Primer name** | **5′–3′ Primers** |
| --- | --- | --- |
| ***ZmCLCg* full length amplification primers** | 77F | TGCTCGTGTCAGTCAGTTCC |
|  | 2713R | ACTTTTACCTTGCCAAGTCGC |
| ***ZmPMP3* full length amplification primers** | 123F | AGCACACGGGTAGAAAAGCA |
|  | 612R | GCAGGGAGGAACGGTTGTAC |
| ***ZmCLCg* target sites amplification primers** | Target1F | GACATCGAGGCCCCGCT |
|  | Target1R | CCACTTGAGCGCCACGTA |
|  | Target2F | GCTTCCTCGTCGGTGCCC |
|  | Target2R | CAATTGTGGTCATGGTTCACG |
|  | Target3F | TTGGTCCTTGGTAACATAGC |
|  | Target3R | GTGAGTGTGTGTTGTCCTATGT |
| **qRT-PCR primers for *ZmCLCg*** | QPCRclcF4 | GACATGTAGGTGGCTCAGGT |
|  | QPCRclcR4 | GGAAAGCAGCAGCAATACCA |
| **qRT-PCR primers for *ZmPMP3*** | QPCRpmpF3 | GCTCGGCTGAAGAAATCAGG |
|  | QPCRpmpR3 | ACAACCCATCAACCCAAGGT |
| **qRT-PCR primers for *ZmActin1*** | 336F | CCCCAAGGCCAACAGAGAGA |
|  | 483R | GCTCACACCATCACCGGAAT |
